# Facile Synthesis of Inorganic Li_2_B_12_H_12_/LiI Solid Electrolytes for High‐Voltage All‐Solid‐State Lithium Batteries

**DOI:** 10.1002/advs.202510193

**Published:** 2025-08-29

**Authors:** Deliang Xu, Mengyuan Jin, Zilong Su, Ran Liu, Mingchuan Xiang, Wanggang Fang, Liqing He, Renbing Wu, Yanhui Guo

**Affiliations:** ^1^ College of Smart Materials and Future Energy Fudan University Shanghai 200433 P. R. China; ^2^ Hefei General Machinery Research Institute Co., Ltd Hefei 230031 P. R. China; ^3^ State Key Laboratory of Coatings for Advanced Equipment Shanghai 200433 P. R. China

**Keywords:** all‐solid‐state lithium batteries, high‐voltage, ionic conduction, Li_2_B_12_H_12_, solid electrolyte

## Abstract

Hydroborate‐based solid electrolytes (SEs), distinguished by their eco‐friendliness and non‐flammability have emerged as a research hotspot in energy storage research. Despite these advantages, their widespread adoption is constrained by insufficient ionic conductivity and poor high‐voltage compatibility. To overcome this challenge, a novel hydroborate SEs, 400‐0.6Li_2_B_12_H_12_‐0.4LiI (denoted as 400‐0.6B_12_‐0.4I), is engineered through a facile synthesis strategy combining high‐temperature processing (400 °C, 2 h, in Ar) and LiI doping. 400‐0.6B_12_‐0.4I exhibits an outstanding ionic conductivity up to 0.75 mS cm^−1^ at 25 °C, which is the highest level among the reported inorganic Li_2_B_12_H_12_‐based SEs so far. It also improves the cycling stability against lithium metal anodes, limits dendrite growth with a high critical current density of 5.0 mA cm^−2^, which enables the Li symmetrical cells to cycle over 1000 h at 25 °C with only 10 mV of overpotential. Moreover, it cycles well in cells using lithium anodes and various high voltage (>4 V vs Li^+^/Li) cathodes materials such as LiCoO_2_, NCM811, and LiMn_2_O_4_. The proposed 400‐0.6B_12_‐0.4I SEs offer valuable guidance for designing next‐generation lithium‐ion solid electrolytes with superior ionic conductivity, enhanced interfacial stability, and exceptional electrode compatibility.

## Graphical abstract

A novel hydroborate‐based solid electrolytes (SEs), 400‐0.6Li_2_B_12_H_12_‐0.4LiI (denoted as 400‐0.6B_12_‐0.4I), all‐solid‐state lithium batteries is developed through facile ball milling process. 400‐0.6B_12_‐0.4I exhibits the highest ionic conductivity among the reported inorganic Li_2_B_12_H_12_‐based SEs so far. It also cycles well with Li anode over 1000 h and compatible with various high voltage cathodes using Li_3_InCl_6_ layer. The proposed 400‐0.6B_12_‐0.4I SEs offer valuable guidance for designing next‐generation all‐solid‐state lithium batteries.

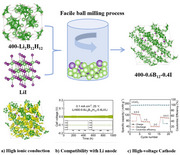

## 1 Introduction

The increasing demand for next‐generation energy‐storage technologies has driven considerable exploration in all‐solid‐state lithium‐metal batteries (ASSLMBs).^[^ ^1^, ^2^, ^3^ ^]^ ASSLMBs are expected to enhance the energy density and safety beyond the limits of present Li‐ion batteries with flammable liquid organic electrolytes. The critical part of ASSLMBs is solid electrolytes (SEs) that promotes rapid ion transport comparable to that of liquid electrolyte. Currently, the most widely studied solid electrolytes are polymer, sulfide, oxide, and halide solid electrolytes, which possess their own advantages and disadvantages. Polymer electrolytes^[^ ^4^ ^]^ are easy of processing but their Li‐ion conductivity and electrochemical stability are not enough to satisfy the requirement of battery technology.^[^ ^5^ ^]^ Sulfide electrolytes can reach high Li‐ion conductivities at room temperatures but there are still safety concerns due to their poor chemical stability.^[^ ^6^, ^7^, ^8^ ^]^ Oxide electrolytes have good electrochemical stability, but the brittleness makes them difficult to adapt to the cathode material volume during the charge/discharge process.^[^ ^9^, ^10^ ^]^ Halide electrolytes have good oxidation stability, but their poor compatibility with lithium metal limits their application in ASSLMBs.^[^ ^11^ ^]^ Despite extensive research efforts in solid‐state battery development,^[^ ^12^, ^13^, ^14^ ^]^ currently no single solid electrolyte has demonstrated holistically optimal characteristics combining high ionic conductivity, exceptional electrochemical stability, and sufficient mechanical robustness to satisfy the operational demands of advanced ASSLMBs.

In order to meet the demands of all‐solid‐state battery applications, we need to develop new solid electrolytes with high ionic conductivity, broad electrochemical stability window, low interface impedance, and good safety. Among the various types of solid electrolyte candidates, hydroborate solid electrolytes^[^ ^15^, ^16^, ^17^, ^18^, ^19^, ^20^, ^21^ ^]^ have attracted considerable interests due to their outstanding performances. The newly discovered boron cage solid electrolytes^[^ ^22^ ^]^ not only have the flexibility and low weight density of conventional borohydride solid electrolytes, but also further enhance the chemical stability and broaden the electrochemical window. Among them, the Li_2_B_12_H_12_ compound has a unique rotatable large‐size B_12_H_12_^2−^ anion structure that facilitates the transport of lithium ions, resulting in high ionic conductivity. This role of accelerating lithium ion transport through the rotational motion of the anion groups has been termed as the paddle‐wheel effect.^[^ ^23^, ^24^ ^]^ In 2015,^[^ ^25^ ^]^ Atsushi Unemoto et al. reported the formation of a stable interface between TiS_2_ and LiBH_4_ in all‐solid‐state lithium‐ion batteries due to the generation of highly stable hydrogen‐deficient Li_2_B_12_H_12_. In 2016,^[^ ^26^ ^]^ they further assembled an all‐solid‐state battery using Li_2_B_12_H_12_ as the solid electrolyte and TiS_2_ as the cathode, and found that its performance was better than that of the battery based on LiBH_4_. In addition, the Li_2_B_12_H_12_ has higher antioxidant properties than LiBH_4_. Nevertheless, the ionic conductivity of Li_2_B_12_H_12_ remains relatively low (≈10^−8^ mS cm^−1^) at RT in comparison to other types of solid electrolytes, which is insufficient for application in ASSLMBs. To further enhance the ionic conductivity of Li_2_B_12_H_12_, it is possible to achieve optimal modification of Li_2_B_12_H_12_ compounds through a range of chemical design strategies, such as effect of anion chemistry.^[^ ^27^, ^28^ ^]^ The introduction of more polarizable and larger anionic compounds into solid‐state electrolyte systems typically results in higher ionic conductivity and lower activation energy.^[^ ^29^ ^]^ We can also disrupt the lithium vacancy order by introducing cationic component,^[^ ^30^, ^31^, ^32^ ^]^ mechanical ball milling, or high‐temperature phase engineering (targeted formation of metastable phases via rapid thermal processing).^[^ ^33^, ^34^ ^]^ to make the solid electrolyte disordered, which broadens the range of lithium site energies and realizes low‐energy migration paths with more connected cages to further improve Li‐ion conductivity. Although the ionic conductivity can be improved to a certain extent, Li_2_B_12_H_12_‐based solid‐state electrolytes have never been significantly improved in terms of suitability for high‐voltage cathode materials. At present, the operating voltage of all‐solid‐state lithium batteries based on inorganic Li_2_B_12_H_12_‐based solid‐state electrolytes is almost always below 4 V.^[^ ^26^, ^35^, ^36^, ^37^, ^38^, ^39^ ^]^ For instance, Li_4_B_10_H_10_B_12_H_12_^[^ ^38^ ^]^ exhibits an operating voltage range of 1.6 to 2.5 V and 2.0 to 3.6 V, respectively, when utilized with titanium disulfide (TiS_2_) or lithium iron phosphate (LiFePO_4_) as the cathode active material. LiF‐decorated Li_2_B_12_H_12_^[^ ^37^ ^]^ exhibits an operating voltage range of 2.1 to 4.0 V with lithium iron phosphate (LiFePO_4_) as the cathode active material. To date, there is hardly Li_2_B_12_H_12_‐based solid‐state electrolyte has been developed that is compatible with high‐voltage cathode materials. Even with sophisticated multi‐component organic modification strategies, the maximum operating voltage of Li_2_B_12_H_12_‐based electrolytes remains constrained at 3.8 V,^[^ ^40^, ^41^ ^]^ thereby restricting its practical utilization in all‐solid‐state batteries. Similar to the dendrite issues in zinc anodes of ZIBs,^[^ ^42^, ^43^ ^]^ lithium dendrite penetration remains a critical challenge in ASSLMBs. The interface engineering strategies developed for zinc anodes^[^ ^44^, ^45^ ^]^ provide valuable insights for our anode/SSE interface design.

Based on the prospective applications of the novel Li_2_B_12_H_12_ SEs in all‐solid‐state lithium‐metal batteries and the inherent limitation of low ionic conductivity and poor high‐voltage compatibility, in this work, we sought to enhance the ionic conductivity of Li_2_B_12_H_12_ through chemical modification, and further verified the performance in ASSLMBs. In detail, the Li_2_B_12_H_12_ was subjected to a high‐temperature quenching treatment first. Then, the high‐temperature treated Li_2_B_12_H_12_ (HT‐Li_2_B_12_H_12_) was further ball milled with lithium halides by high‐energy ball milling to get the composite electrolyte. Excitingly, the anion‐mixed 400‐0.6Li_2_B_12_H_12_‐0.4LiI (denoted as 400‐0.6B_12_‐0.4I) synthesized through co‐ball‐milling of Li_2_B_12_H_12_ (pre‐treated at 400 °C) and LiI in a molar ratio of 0.6:0.4 exhibits ionic conductivity up to 0.75 mS cm^−1^ at room temperature, which is three orders of magnitude higher than that of Li_2_B_12_H_12_. To understand the mechanism of lithium ion conduction, we have analysed the structure and composition of the solid‐state electrolyte and performed theoretical calculations. The results show that by high temperature treatment of Li_2_B_12_H_12_ and ball milling with LiI, the structure of SEs becomes disordered, which favours lithium ion conduction. 400‐0.6B_12_‐0.4I also lowers the interfacial resistance with Li metal, improves the cycling stability in symmetric cells with Li electrodes, and serves as a stable separator in cells using a series of high voltage cathodes materials such as LiCoO_2_ cathodes, NCM811 cathodes, and LiMn_2_O_4_ cathodes. This work demonstrates that 400‐0.6B_12_‐0.4I is a significant SEs candidate and lays a good foundation for future studies of modifying Li_2_B_12_H_12_ for high‐energy density ASSLMBs.

## 2 Results and Discussion

**Figure 1:**
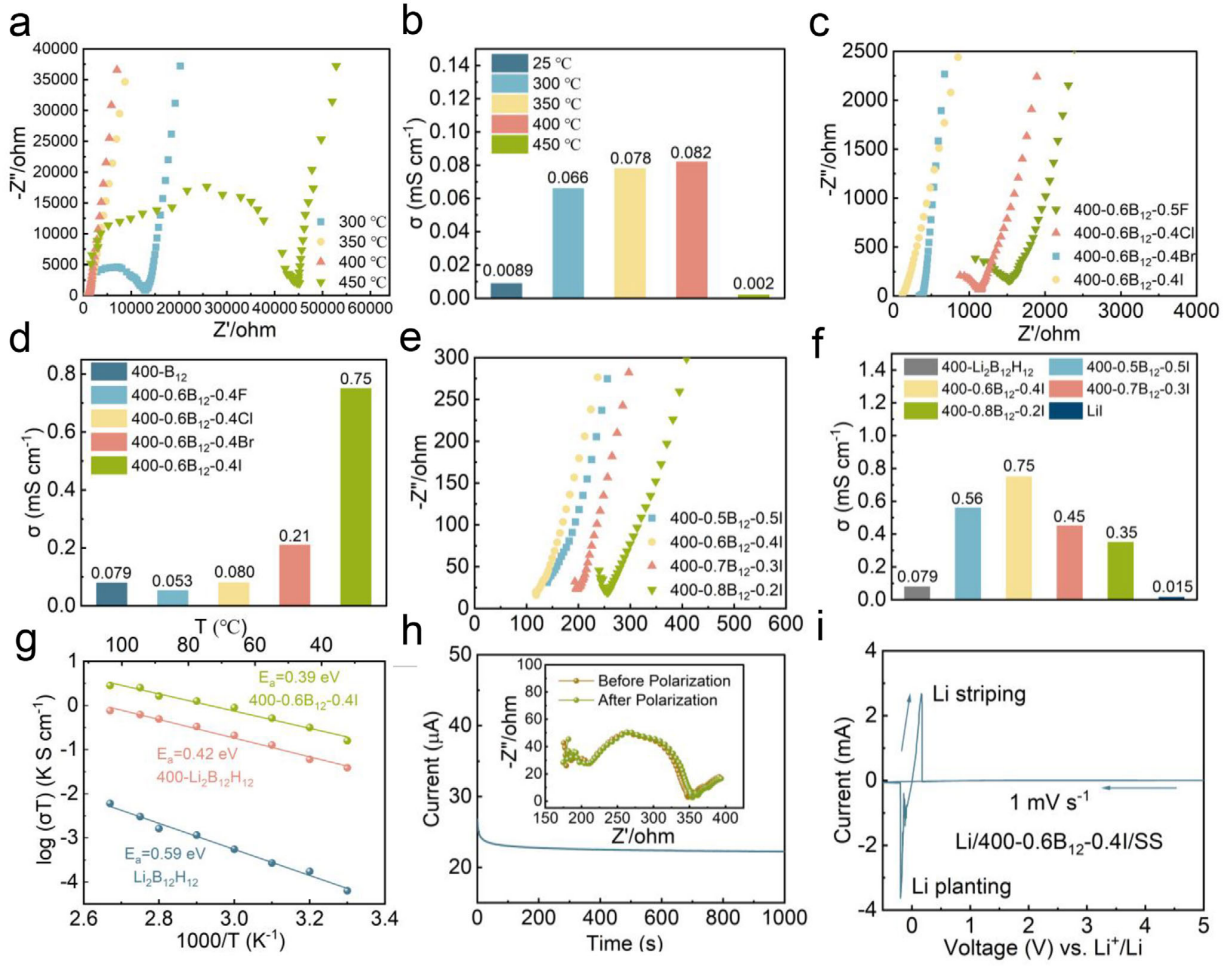
Li_2_B_12_H_12_ based SEs electrochemical evaluation. a) EIS of Li_2_B_12_H_12_ sintered at various temperature. b) Ionic conductivities of Li_2_B_12_H_12_ sintered at various temperature. c) EIS of 400‐0.4B_12_‐0.6X (X= F, Cl, Br, I). d) Ionic conductivities of 400‐0.4B_12_‐0.6X (X= F, Cl, Br, I). e) EIS of 400‐xB_12_‐yI. f) Ionic conductivities of 400‐xB_12_‐yI. g) Arrhenius plots of of pure Li_2_B_12_H_12_, 400‐Li_2_B_12_H_12_ and 400‐0.6B_12_‐0.4I with activation energies (Ea) of 0.59, 0.42, and 0.39 eV, respectively. h) Chronoamperometry profiles and electrochemical impedance spectra (inset) before and after polarization of a symmetric Li/400‐0.6B_12_‐0.4I/Li block cell with a polarization voltage of 10 mV. i) LSV curve of Li/400‐0.6B_12_‐0.4I/SS with a scan rate of 1 mV s^−1^ at 25 °C.

In this study, we report a dual optimization strategy for Li_2_B_12_H_12_ involving controlled thermal annealing protocols and lithium halide (LiX) incorporation. First, the Li_2_B_12_H_12_ was synthesized via a cation exchange method using (Et_3_NH)_2_(B_12_H_12_) as the precursor (Figure S1a, Supporting Information), following an optimized literature protocol.^[^ ^46^ ^]^ The crystalline structure of the synthesized material was systematically characterized by X‐ray diffraction (XRD) analysis (Figure S1b, Supporting Information), two distinct diffraction peaks at 15.9° and 18.4°, corresponding to the (111) and (200) crystallographic planes respectively, confirm the successful formation of phase‐pure Li_2_B_12_H_12_. Further structural validation was obtained through solution‐state nuclear magnetic resonance (NMR) spectroscopy, where both ^11^B NMR and ^1^H NMR spectra (Figure S2, Supporting Information) exhibit characteristic resonance patterns consistent with the anticipated borohydride structure, thereby providing complementary evidence for the complete cation substitution and preservation of the B_12_H_12_^2−^ cluster integrity during the synthesis process. A series of modified Li_2_B_12_H_12_ materials were further synthesized through systematic variation of calcination temperatures (300, 350, 400, 450 °C) at argon atmosphere, designated as 300‐Li_2_B_12_H_12_, 350‐Li_2_B_12_H_12_, 400‐Li_2_B_12_H_12_, and 450‐Li_2_B_12_H_12,_ respectively. The effects of different sintering temperatures on the electrochemical impedance spectroscopy (EIS) of Li_2_B_12_H_12_ were studied (**Figure** **1**a). The EIS results demonstrate that when the temperature is below 400 °C, the impedance of Li_2_B_12_H_12_ SEs decreases significantly with the increase of temperature. However, the impedance of Li_2_B_12_H_12_ increases substantially at 450 °C, suggesting the onset of structural instability or decomposition phenomena at elevated temperatures. The corresponding ionic conductivity results also show that the 400‐Li_2_B_12_H_12_ exhibited higher ionic conductivity (0.082 mS cm^−1^ at 400 °C) than pristine Li_2_B_12_H_12_ (0.0089 mS cm^−1^ at 25 °C), while the ionic conductivity of 450‐Li_2_B_12_H_12_ (0.02 mS cm^−1^ at 400 °C) was lower than the pristine Li_2_B_12_H_12_ (Figure 1b). The thermal activation process at a certain degree of heat treatment results in a substantial improvement in ionic conductivity compared to room‐temperature values. However, a notable conductivity degradation occurs when the sintering temperature reaches 450 °C. Building upon the optimized thermal processing at 400 °C, we implemented a strategic lithium halide (LiX) doping protocol to enhance the ionic transport properties of 400‐Li_2_B_12_H_12_. The mechanochemical synthesis of composite solid electrolytes was achieved through high‐energy ball milling of 400‐Li_2_B_12_H_12_ with LiX (X═F, Cl, Br, I) at controlled stoichiometric ratios (0.6:0.4 molar ratio), yielding the 400‐0.6B_12_‐0.4X (X═F, Cl, Br, I) series. Electrochemical impedance spectroscopy (EIS) characterization (Figure 1c) revealed a halide‐dependent conductivity enhancement mechanism: while 400‐0.6B_12_‐0.4F incorporation decreased ionic conductivity (0.053 mS cm^−1^), 400‐0.6B_12_‐0.4Cl (0.080 mS cm^−1^), 400‐0.6B_12_‐0.4Br (0.21 mS cm^−1^), and 400‐0.6B_12_‐0.4I (0.75 mS cm^−1^) demonstrated progressively improved performance (Figure 1d). This trend correlates strongly with the ionic radius‐polarizability relationship (F^−^: 1.33 Å, Cl^−^: 1.81 Å, Br^−^: 1.96 Å, I^−^: 2.20 Å). Larger halogen ion doping synergistically enhances the conductivity of lithium ions through the following mechanisms: 1, enlarging the size of ion transport channels to reduce the migration constraints and electrostatic repulsion; 2, inducing greater lattice distortion that facilitates Frenkel defect formation and lithium ion mobility through crystal spatial structure, disrupting the locally ordered structure to increase the lithium ion‐occupiable sites; 3, altering the cation coordination environment to weaken the electrostatic interaction; and 4, optimizing the grain boundary properties to reduce the grain boundary resistance. Together, these structural modifications reduce the lithium ion migration energy barrier and significantly enhance the overall ionic conductivity. Based on the good ionic conductivity properties of 400‐Li_2_B_12_H_12_ combined with LiI ball milling, we further optimized the doping concentration of LiI in four stoichiometries (20–50 mol%): 400‐0.8B_12_‐0.2I, 400‐0.7B_12_‐0.3I, 400‐0.6B_12_‐0.4I, 400‐0.5B_12_‐0.5I, which revealed the 400‐0.6B_12_‐0.4I show the lowest impedance in all the doping concentration of LiI (Figure 1e). The 400‐0.6B_12_‐0.4I composition exhibited peak ionic conductivity (0.75 mS cm^−1^ at 25 °C) (Figure 1f), representing an 84‐fold enhancement over pristine Li_2_B_12_H_12_ (0.0089 mS cm^−1^ at 25 °C), which is at a significantly leading level among borohydride solid electrolytes. (Table S1, Supporting Information). The Nyquist plots of 400‐0.6B_12_‐0.4I with the equivalent circuit model are shown in Figure S3 (Supporting Information). The fitted Rb and Rgb value in the proposed equivalent circuit model was used to determine the bulk resistance and grain boundary resistance. Based on the fitted results, it can be seen that the solid‐state electrolyte has a bulk impedance of 118.12 Ω and a grain boundary impedance of 13.9 Ω, indicating that LiI enhances ionic conductivity not only by doping into 400‐Li_2_B_12_H_12_, but also by improving the interfacial impedance. Based on the good ionic conductivity of 400‐0.6B_12_‐0.4I, we conducted a subsequent series of characterizations around 400‐0.6B_12_‐0.4I to verify its potential for application in all‐solid‐state lithium batteries. Arrhenius analysis demonstrated significant activation energy reduction from 0.59 eV (pristine Li_2_B_12_H_12_) to 0.39 eV (400‐0.6B_12_‐0.4I), indicating modified lithium migration pathways with lower energy barriers (Figure 1g). Complementary DC polarization measurements confirmed near‐unity Li⁺ transference number (t_Li⁺_ = 0.94), confirming the domination of Li‐ion conduction (Figure 1h). The electronic conductivity of 400‐0.6B_12_‐0.4I was calculated to be negligibly low 3.07 × 10^−11^ S cm^−1^, the low electronic conductivity of 400‐0.6B_12_‐0.4I significantly reduces the formation of lithium dendrite compounds, this helps to maintain a favorable battery lifetime and performances (Figure S4, Supporting Information). Voltage windows of 400‐0.6B_12_‐0.4I were tested by cyclic voltammetry (CV) measurement; the measurement started from the −0.5 to 5.0 V, then to −0.5 V, which was returned to the OCV. The results show that there is no obvious other peak in addition to Li's oxidation reduction peak, which proves that 400‐0.6B_12_‐0.4I displayed superior electrochemical reduction stability (Figure 1i), thereby ensuring optimal compatibility with lithium metal anodes.

**Figure 2:**
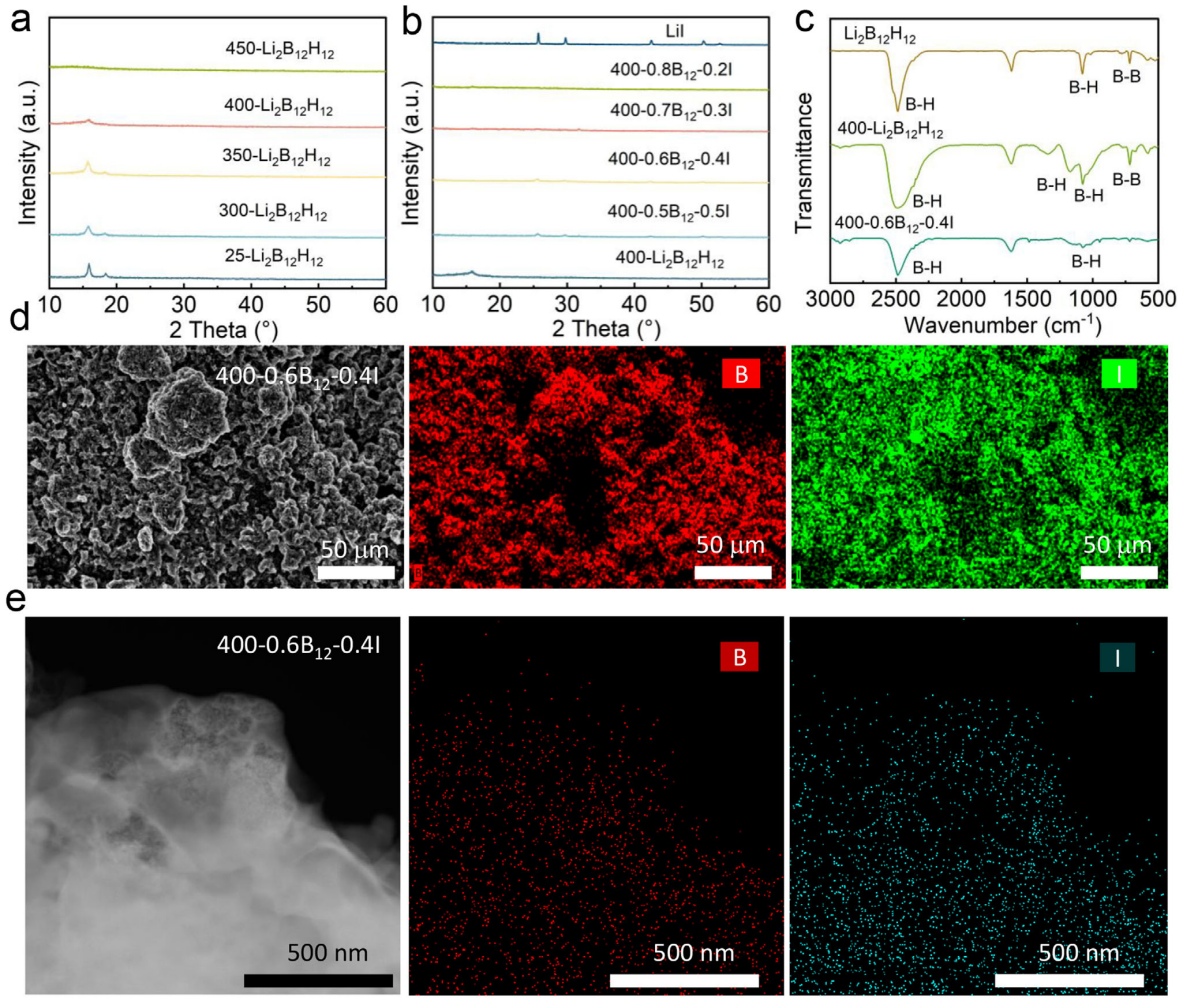
Structural and morphological characterization of Li_2_B_12_H_12_‐based SEs. a) Temperature‐dependent X‐ray diffraction (XRD) patterns of Li_2_B_12_H_12_ during sintering treatment. b) Composition‐dependent XRD analysis of 400‐xB_12_‐yI with varying stoichiometric ratios. c) Comparative Fourier‐transform infrared (FTIR) spectroscopy of pristine Li_2_B_12_H_12_, thermally processed Li_2_B_12_H_12_ (400‐Li_2_B_12_H_12_), and optimized composite material (400‐0.6B_12_‐0.4I) d) SEM image of 400‐0.6B_12_‐0.4I with EDS mapping of boron and iodine. e) TEM image of 400‐0.6B_12_‐0.4I and corresponding EDS mapping of boron and iodine.

In order to understand the mechanism of 400‐0.6B_12_‐0.4I ion conduction, the structural evolution and compositional characteristics of novel solid‐state electrolytes, synthesized through high‐temperature processing of Li_2_B_12_H_12_ followed by lithium halide complexation, were systematically investigated. Closo‐borate compounds typically exhibit polycrystalline phase transitions upon thermal activation, with Li_2_B_12_H_12_ demonstrating a well‐documented order‐disorder transition at 355 °C, progressing from a low‐temperature face‐centered cubic (fcc) ordered configuration to a high‐temperature structurally disordered state.^[^ ^38^ ^]^ Our XRD analysis of Li_2_B_12_H_12_ samples prepared under varied thermal conditions (**Figure** **2**a) revealed temperature‐dependent structural modifications, the results showed that the characteristic peak of Li_2_B_12_H_12_ gradually weakened with the increase of temperature, and finally disappeared completely at 450 °C. The XRD experimental results confirm the transition from ordered to disordered structure of Li_2_B_12_H_12_ by high‐temperature treatment, which is favorable for Li^+^ conduction. Furthermore, after ball milling of 400‐Li_2_B_12_H_12_ with LiI in different ratios, the degree of XRD crystallinity was further weakened, and the structure became amorphous (Figure 2b). This dual‐modification strategy (thermal disordering coupled with mechanical amorphization) establishes percolation pathways through disrupted boron cluster arrangements and optimized halide distribution, fundamentally explaining the observed order‐of‐magnitude enhancement in ionic conductivity (from 0.0089 to 0.75 mS cm^−1^). The structure‐property correlation underscores the critical role of controlled disorder engineering in developing high‐performance borate‐based solid electrolytes. In addition, the FT‐IR results (Figure 2c) show that Li_2_B_12_H_12_, 400‐Li_2_B_12_H_12_, and 400‐0.6B_12_‐0.4I have obvious B─H stretching vibration peaks at 2500–2600 cm^−1^, Li_2_B_12_H_12_ has relatively weak B‐H bending vibration peak at 1100 cm^−1^, and the B─H bending vibration peak of 400‐Li_2_B_12_H_12_ is broadened and split, reflecting the change of local bonding environment. The B─H bending vibration peak of 400‐Li_2_B_12_H_12_ is broadened and split, reflecting the change of the local bond environment, which is mainly due to the disordered structure of Li_2_B_12_H_12_ with a small amount of hydrogen released from the high‐temperature treatment.^[^ ^47^, ^48^ ^]^ The B─B bond vibration shows weaker absorption peaks in the low‐wave number region (500‐800 cm^−1^), indicating that the B─H bending vibration peaks of 400‐0.6B_12_‐0.4I and the B─B bond vibration peaks gradually disappeared, which may be due to the weaker peak intensity, and the peaks were masked after co‐ball milling with LiI. We further analyzed the structures of Li_2_B_12_H_12_, 400‐Li_2_B_12_H_12_ and 400‐0.6B_12_‐0.4I SEs by XPS (Figure S5, Supporting Information), and the results show that Li_2_B_2_H_12_ after high temperature treatment at 400 °C still maintains the boron cage structure, and the B peaks broaden or split, reflecting the coexistence of boron atoms in multiple chemical environments, which may be the result of the combined effect of partial dehydrogenation and structural distortion.^[^ ^49^, ^50^ ^]^ This may be a combination of partial dehydrogenation and structural distortion. Meanwhile, due to the dehydrogenation effect, the loss of H may lead to the decrease of B─H bond, the increase of B─B bond ratio, the decrease of electron density, and the small increase of binding energy. The XPS results of 400‐Li_2_B_12_H_12_ show the presence of LiI, which confirms that LiI are successfully complexed and doped in 400‐Li_2_B_12_H_12_. The Raman results show that Li_2_B_12_H_12_ exhibits obvious B─B skeletal vibration peaks around 750 cm^−1^ and B‐H telescopic vibration peaks around 2500 cm^−1^ at room temperature. The B‐B and B─H Raman peaks are significantly weaker when treated at 400 °C, mainly because the Li_2_B_12_H_12_ crystal structure undergoes a phase transition to a more disordered after high‐temperature treatment. The 400‐0.6B_12_‐0.4 solid‐state electrolyte exhibits B─B and B─H Raman characteristic peaks, as well as I‐I peaks, which further confirms the incorporation of LiI (Figure S6, Supporting Information). Additionally, the DSC results showed that Li_2_B_12_H_12_ exhibits significant heat absorption peaks at 170 and 342 °C, indicating a transition from an ordered phase at low temperatures to a disordered phase. Additionally, an exothermic peak appears at 556 °C, suggesting the decomposition of the sample. In contrast, the DSC spectrum of 400‐0.6B_12_‐0.4I exhibits broad peak which mainly arises from its amorphous structure, this structural feature means the 400‐0.6B_12_‐0.4I undergoes a continuous thermal transition rather than exhibiting a distinct phase transition temperature. Consequently, energy absorption or release occurs over a broad temperature range during heating or cooling, reflected as a broad DSC peak (Figure S7, Supporting Information). The weight loss of 400‐0.6B_12_‐0.4I in the TG/MS experiment is 6.4%, and the weight loss of Li_2_B_12_H_12_ is 9.2%. On the one hand, due to the fact that both Li_2_B_12_H_12_ and 400‐0.6B_12_‐0.4I decompose at high temperature by releasing H_2_, the high temperature treatment at 400 °C as well as the introduction of lithium iodide elevated the decomposition temperature by 30.8 °C, which significantly optimized the thermal stability (Figure S8, Supporting Information).

The SEM photographs show that the grain size of Li_2_B_12_H_12_ increases significantly after high‐temperature heat treatment at 400 °C. The grain size of 400‐0.6B_12_‐0.4I prepared by ball milling of Li_2_B_12_H_12_ together with LiI decreases significantly, and the densification is improved compared with Li_2_B_12_H_12_, which is to some extent favourable for Li^+^ conduction (Figure S9, Supporting Information). The EDS mapping of 400‐0.6B_12_‐0.4I shows homogeneous B signals and I signals distribution, which can be attributed to the distribution of LiI and 400‐Li_2_B_12_H_12_. The I signals are observed around grain boundaries and within the polycrystals grains, suggesting that LiI doped into the interior of the 400‐Li_2_B_12_H_12_ grains (Figure 2d). TEM elemental mapping also shows significant B and I signals, further confirming I‐ion entry into the lattice of 400‐Li_2_B_12_H_12_, which facilitates the conduction of lithium ions (Figure 2e). The 400‐0.6B_12_‐0.4I SEs demonstrated a remarkably low true density of 1.541 g cm^−3^ as determined by a true density analyser, representing one of the lightest reported values among solid electrolyte systems to date (Figure S10, Supporting Information). Low‐density 400‐0.6B_12_‐0.4I SEs not only could improve processability for flexible designs and better electrode contact, but also have higher mass energy density by enabling thinner layers or more electrode material per volume.

**Figure 3:**
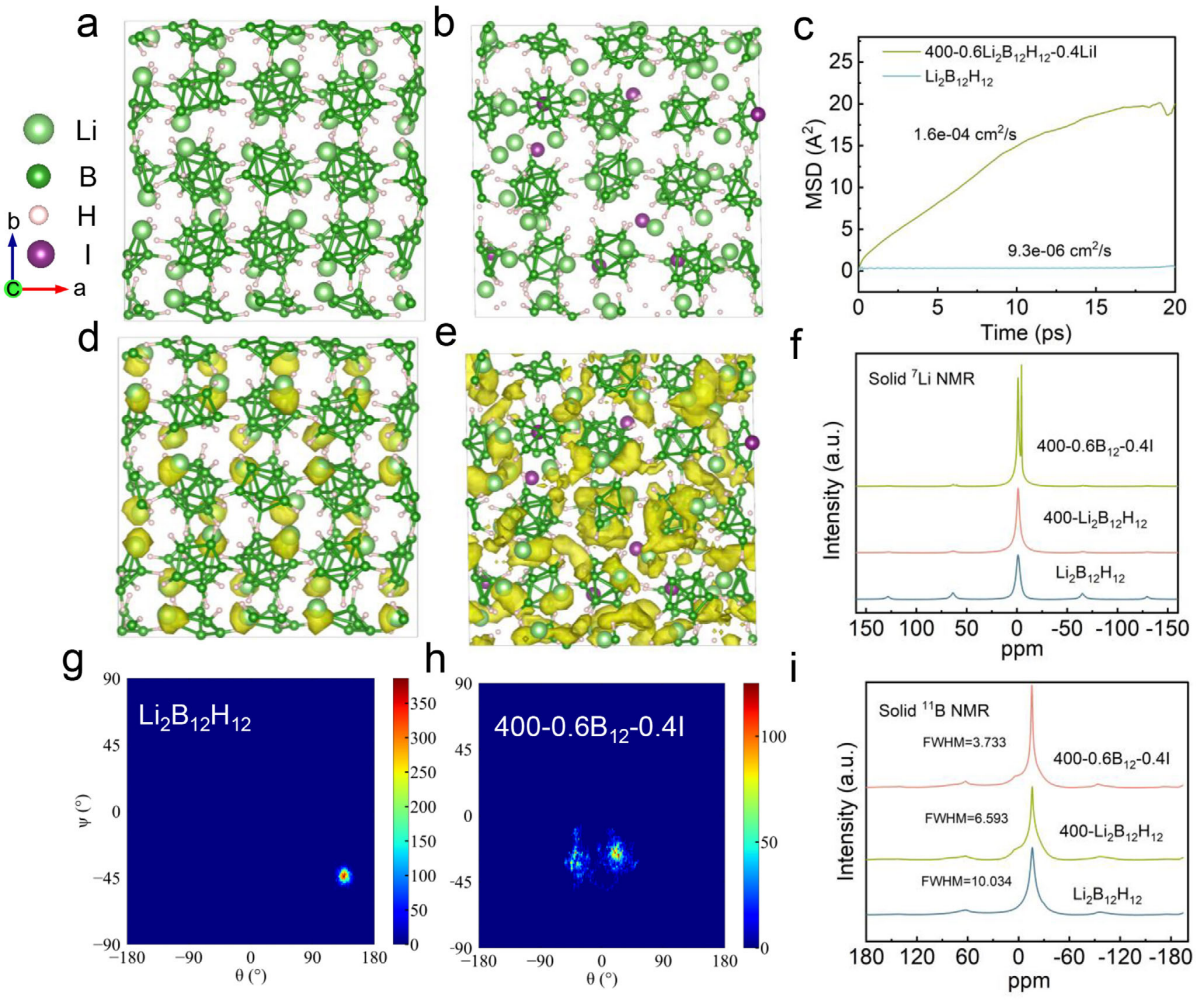
Mechanistic investigation of lithium‐ion conduction in 400‐0.6B_12_‐0.4I SEs. a,b) Proposed structural models of Li_2_B_12_H_12_ and 400‐0.6B_12_‐0.4I, illustrating structural modifications induced by LiI integration. c) The mean square displacement (MSD) profiles of Li_2_B_12_H_12_ and 400‐0.6B_12_‐0.4I with calculated diffusivity values of 9.3 × 10^−6^ and 1.6 × 10^−4^ cm^2^s^−1^ based on AIMD simulations over 20 ps. d,e) Lithium‐ion probability density map of Li_2_B_12_H_12_ and 400‐0.6B_12_‐0.4I, visualizing ion migration pathways within the boron‐hydrogen framework. f) Solid‐state ^7^Li NMR spectra of Li_2_B_12_H_12_ and 400‐0.6B_12_‐0.4I. g,h) Angular preudo‐density of a representative B_12_H_12_ group in the structure of Li_2_B_12_H_12_, 400‐Li_2_B_12_H_12_, and 400‐0.6B_12_‐0.4I, revealing structural reorientation effects from LiI incorporation. i) Solid‐state ^11^B NMR spectra contrasting boron coordination states of Li_2_B_12_H_12_, 400‐Li_2_B_12_H_12_, and 400‐0.6B_12_‐0.4I.

In order to thoroughly investigate the effect of I‐ion doping on the ionic conductivity of 400‐0.6B_12_‐0.4I, density functional theory (DFT) and ab initio molecular dynamics (AIMD) simulations were performed to investigate the Li^+^ diffusion mechanism underlying the improved conductivity of 400‐0.6B_12_‐0.4I. To simplify the calculation process, we mainly analysed the effect of LiI doping on the ionic conductivity of Li_2_B_12_H_12_ treated at high temperature. The face‐centered cubic structure crystalline Li_2_B_12_H_12_ (**Figure** **3**a) and amorphous 400‐0.6B_12_‐0.4I (Figure 3b) were simulated for 20 ps at 327 °C to study the Li ion diffusion processes using AIMD simulation.^[^ ^51^ ^]^ The diffusion of lithium ions in 400‐0.6B_12_‐0.4I and Li_2_B_12_H_12_ was quantified by the mean squared displacements (MSD). The MSD of Li with time represents the diffusion of Li^+^ in the Li_2_B_12_H_12_ and 400‐0.6B_12_‐0.4I (Figure 3c), the slope of the MSD curve for 400‐0.6B_12_‐0.4I represents the diffusion of Li ions (1.6 × 10^−4^ cm^2^s^−1^ at 400 °C), which is much higher than untreated Li_2_B_12_H_12_ (9.3 × 10^−6^ cm^2^s^−1^ at 400 °C). The MSD results demonstrated the much faster diffusion of Li^+^ in the 400‐0.6B_12_‐0.4I materials than the Li_2_B_12_H_12_.

In addition, the results of the AIMD simulations show that the Li^+^ in Li_2_B_12_H_12_ are unable to form an interconnected Li^+^ diffusion network and can only fluctuate around the normal thermal vibration of their equilibrium position (Figure 3d). The amorphous 400‐0.6B_12_‐0.4I shows greater dispersion density at the same isosurface level, suggesting that Li^+^ diffusion in 400‐0.6B_12_‐0.4I SE is significantly enhanced to form a 3D lithium ion diffusion network (Figure 3e). To further investigate Li^+^ transport in the 400‐0.6B_12_‐0.4I SE, we used solid‐state nuclear magnetic resonance (NMR) to analyze the local chemical structure and dynamics (Figure 3f). ^7^Li NMR spectra of 400‐0.6B_12_‐0.4I in the solid state show narrower peak than that of Li_2_B_12_H_12_, indicating faster Li ion dynamics in the 400‐0.6B_12_‐0.4I.^[^ ^52^ ^]^ Meanwhile, solid‐state NMR results show a new peak in the lithium spectrum of 400‐0.6B_12_‐0.4I, which is mainly attributed to the LiI composite modification. The ^7^Li NMR experimental results are in good agreement with the extended 3D Li ion conduction paths and higher Li^+^ MSDs in 400‐0.6B_12_‐0.4I derived from AIMD simulations. We further studied the reorientation of B_12_H_12_^2−^ through calculating the angular pseudodensity on the basis of the binning of (*θ, ψ*) values (Figure S11, Supporting Information). Single angular pseudodensity maps of Li_2_B_12_H_12_ (Figure 3g) and 400‐0.6B_12_‐0.4I (Figure 3h) were chosen for comparison under the same conditions. In comparison to Li_2_B_12_H_12_, the B_12_H_12_ units in 400‐0.6B_12_‐0.4I exhibit a greater propensity for rotation within a larger region of high density, which may facilitate Li‐ion diffusion based on the paddle‐wheel mechanism.^[^ ^23^ ^]^ Similarly, the full width at half maxima (FWHM) of the solid ^11^B NMR spectrum of 400‐0.6B_12_‐0.4I is calculated to be 3.733, which is much lower than the FWHM of Li_2_B_12_H_12_ (10.034), reflecting the rapid dynamics of the [B_12_H_12_]^2−^ polyanion of 400‐0.6B_12_‐0.4I (Figure 3i), this further indicates that 400‐0.6B_12_‐0.4I has a more noticeable paddle‐wheel effect. Tracking the trajectories of individual B and Li atoms by AIMD calculations reveals that the B and Li atoms of 400‐0.6B_12_‐0.4I have more pronounced trajectories than those of Li_2_B_12_H_12_. The B‐atoms show a clear enhancement of the vibrational amplitude near their equilibrium position, which promotes the motion of the lithium ions (Figure S12, Supporting Information).

**Figure 4:**
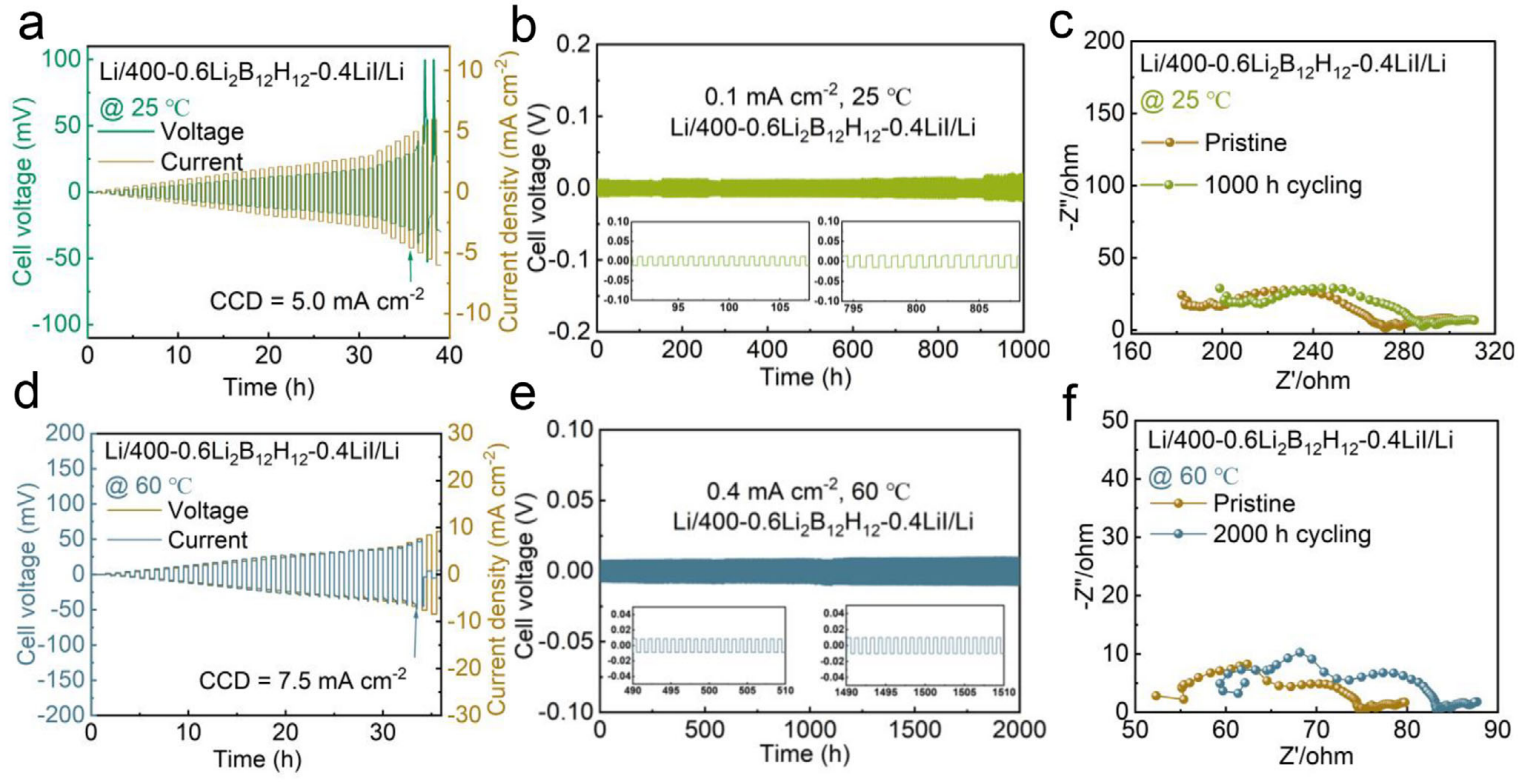
Electrochemical performance and anode analysis of Li/400‐0.6B_12_‐0.4I/Li symmetric cells at 25 and 60 °C. a) Critical current density (CCD) evaluation of a Li/400‐0.6B_12_‐0.4I /Li symmetric cell with step‐increased current densities at 25 °C. b) Long‐term galvanostatic cycling profile of the symmetric cell at 25 °C under a current density of 0.1 mA cm^−2^, highlighting stable lithium plating/stripping over 1000 hours. c) Comparative AC impedance spectra of Li/400‐0.6B_12_‐0.4LiI /Li cell before and after 1000 h cycling at 25 °C, illustrating interfacial resistance evolution. d) Elevated‐temperature CCD testing of a Li/400‐0.6B_12_‐0.4I/Li symmetric cell with step‐increased current densities at 60 °C, revealing enhanced CCD under thermal activation. e) Long‐term galvanostatic cycling profile of the symmetric cell under a current density of 0.4 mA cm^−2^ at 25 °C. f) the corresponding AC impedance spectra of Li/400‐0.6B_12_‐0.4I /Li cell before and after 2000 h cycling at 60 °C.

Given the superior ionic transport properties of 400‐0.6B_12_‐0.4I SEs, we systematically studied their potential applications in all‐solid‐state lithium metal battery systems. We initially investigated the compatibility between the 400‐0.6B_12_‐0.4I SEs and lithium anode. The 400‐0.6B_12_‐0.4I SEs exhibits remarkable reductive stability and exceptionally low electronic conductivity (3.07 × 10^−11^ S cm^−1^), suggesting superior compatibility with lithium metal anodes. To systematically evaluate the lithium metal anodes compatibility of 400‐0.6B_12_‐0.4I SEs, we constructed symmetric Li/400‐0.6B_12_‐0.4I/Li cell. The critical current density of Li/400‐0.6B_12_‐0.4I/Li can reach 5 mA cm^−2^ at 25 °C (**Figure** **4**a), indicative of robust dendrite suppression capabilities of 400‐0.6B_12_‐0.4I SEs. Furthermore, the long‐term cycling stability test of the symmetric cell was conducted at 0.1 mA cm^−2^ and displays a flat Li plating/stripping voltage profile for more than 1000 h without short circuiting (Figure 4b). Electrochemical impedance spectroscopy of the Li/400‐0.6B_12_‐0.4I/Li cell revealed minimal changes in interfacial resistance after cycling 1000 h, which is correlating well with the observed slight increase in overpotential (Figure 4c). Consistent with our expectations, the 400‐0.6B_12_‐0.4I SEs exhibits remarkable interfacial compatibility and electrochemical stability when paired with the lithium metal anode. We further explored the effect of 400‐0.6B_12_‐0.4I SEs on the performance of lithium‐symmetric batteries under high‐temperature conditions at 60 °C, which yielded enhanced performance metrics. The results show that the critical current density Li/400‐0.6B_12_‐0.4I/Li can be increased to 7.5 mA cm^−2^ when the temperature is increased to 60 °C (Figure 4d). The Li/400‐0.6B_12_‐0.4I/Li also shows the stable Li plating and stripping for 2000 h at 0.4 mA cm^−2^ (Figure 4e). The impedance resistances of Li/400‐0.6B_12_‐0.4I/Li symmetric batteries were also measured before and after 2000 h cycles at 60 °C, which shows slightly increase (Figure 4f). The experimental results of symmetrical battery show that 400‐0.6B_12_‐0.4I SEs has good compatibility with lithium metal anode. In Figure 4b,e, the overpotential appears to show an upward trend over time, the increase in overpotential primarily results from microgaps caused by volume changes and the formation of a high‐impedance layer due to the interdiffusion of elements at the electrode/electrolyte interface during cycling, rather than from intrinsic material instability. Microgaps reduce the effective contact area, resulting in uneven current distribution and blocking ion transport. At the same time, uneven local stripping and deposition of the lithium anode create “dead lithium” and holes, which exacerbates local polarization. The loss of interfacial contact and growth of the side reaction layer together lead to an increase in overpotential.

**Figure 5:**
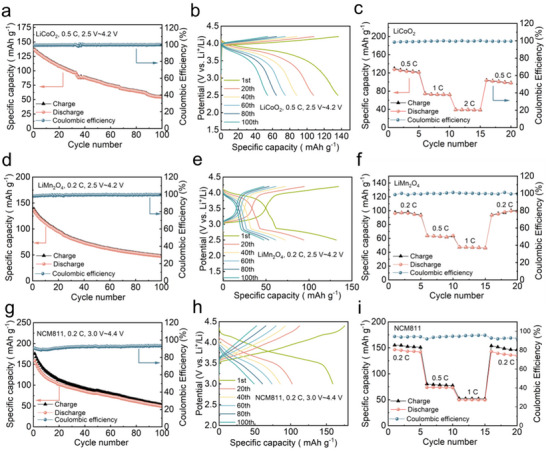
All‐solid‐state battery performance with high voltage cathodes of the 400‐0.6B_12_‐0.4I SE. a) Cycle performance, b) corresponding GDC curves, and c) rate performance of Li/400‐0.6B_12_‐0.4I/Li_3_InCl_6_/LiCoO_2_. d) Cycle performance, e) corresponding GDC curves, and f) rate performance of Li/400‐0.6B_12_‐0.4I/Li_3_InCl_6_/LiMn_2_O_4_. g) Cycle performance, h) corresponding GDC curves, and i) rate performance of Li/400‐0.6B_12_‐0.4I/Li_3_InCl_6_/NCM811.

We further evaluated the performance of 400‐0.6B_12_‐0.4I in a full cell at 60 °C, using a bare lithium metal anode and a LiFePO_4_ cathode. the capacity of Li|400‐0.6B_12_‐0.4I|LiFePO_4_ cell decreased from 137.9 to 115.7 mAh g^−1^ at 0.2 C after 100 cycles, exhibiting a capacity retention of 83.9% as shown in corresponding GDC (Galvanostatic discharge and charge) and cycle performance curves (Figure S13, Supporting Information). According to the experimental results, the cell's capacity inevitably declines after a long period of cycling, which needs to be further improvement in future research. Despite the promising properties of Li_2_B_12_H_12_‐based solid electrolytes (SEs), their compatibility with high‐voltage cathodes such as NCM811, LiCoO_2_, and LiMn_2_O_4_ has remained unexplored. Solid‐state electrolytes based on Li_2_B_12_H_12_ are intrinsically intolerant of high‐voltage operation. The fundamental incompatibility with high‐voltage cathodes (>4.0 V vs. Li⁺/Li) arises from the irreversible oxidative decomposition of their [B_12_H_12_]^2−^ anion cages. This decomposition triggers interfacial failure, increased impedance, capacity decay, and safety risks. When the solid‐state electrolyte is directly matched with NCM811, LiCoO_2_, and LiMn_2_O_4_, its capacity is very low, and the capacity declines significantly, approaching zero in the 10th (Figure S14, Supporting Information). To address this gap, we investigated the feasibility of using 400‐0.6B_12_‐0.4I as an electrolyte in high‐voltage solid‐state batteries. Recent advancements have shown an efficient artificial solid electrolyte interphase (ASEI) protective layer between the cathode and separator can significantly reduce interfacial degradation. This ASEI prevents direct contact and side reactions between Li_2_B_12_H_12_ and the high‐voltage cathode through combined physical isolation and chemical passivation. In sight of these findings, a protective layer of Li_3_InCl_6_ was incorporated between the high‐voltage cathode and 400‐0.6B_12_‐0.4I SEs. Proof‐of‐concept batteries using lithium metal anodes and LiCoO_2_, LiMn_2_O_4_, or NCM811 as the cathode active material (CAM) were assembled in custom pressure cells with a 10 mm diameter active area. The cathode composition included 10 wt.% Super C65 as a conductive additive, with a CAM mass loading of 0.9 mg cm^−2^. When cycled at 0.1 C (1 C = 140 mA g_CAM_^−1^) and 60 °C between 2.5 and 4.2 V, the LiCoO_2_/Li_3_InCl_6_/400‐0.6B_12_‐0.4I/Li cell delivers an initial discharge capacity of 137 mAh g^−1^ with a coulombic efficiency of 99.9% (**Figure** **5**a). Moreover, the LiCoO_2_/Li_3_InCl_6_/400‐0.6B_12_‐0.4I/Li cell showed long‐term cyclability with a discharge capacity retention of 52 mAh g^−1^ after 100 cycles at 0.5 C (Figure 5b). The cell also displays a good rate performance at 0.5 C, 1 C, 2 C (Figure 5c). Similar trends were observed for LiMn_2_O_4_‐based and NCM811‐based cells. The LiMn_2_O_4_/Li_3_InCl_6_/400‐0.6B_12_‐0.4I/Li cell showed initial discharge capacities of 132 mAh g^−1^ when cycled at 0.2 C and 60 °C (Figure 5d). In addition, the LiMn_2_O_4_/Li_3_InCl_6_/400‐0.6B_12_‐0.4I/Li cell demonstrated long‐term cyclability with a discharge capacity retention of 49 mAh g^−1^ after 100 cycles at 0.2 C (Figure 5e). The cell also displays a good rate performance at 0.2, 0.5, and 1 C (Figure 5f).

The NCM811/Li_3_InCl_6_/400‐0.6B_12_‐0.4I/Li cell showed initial discharge capacities of 160 mAh g^−1^ when cycled at 0.2 C and 60 °C (Figure 5g). In addition, the NCM811/Li_3_InCl_6_/400‐0.6B_12_‐0.4I/Li cell showed long‐term cyclability with a discharge capacity retention of 50 mAh g^−1^ after 100 cycles at 0.2 C (Figure 5h). The cell also displays a good rate performance at 0.2, 0.5, and 1 C (Figure 5i). All‐solid‐state battery results confirm that 400‐0.6B_12_‐0.4I SE could match with diverse high‐voltage cathodes when using Li_3_InCl_6_ protective layer, overcoming the traditional voltage limitations of hydroborate electrolytes, which is much better than the other inorganic Li_2_B_12_H_12_‐based solid electrolytes (Table S2, Supporting Information) and demonstrates significant potential for widespread application in the field of all‐solid‐state batteries.^[^ ^52^ ^]^ Although the results show that there is a significant increase in the full cell capacity with NCM811, LiCoO_2_, and LiMn_2_O_4_ when the Li_3_InCl_6_ protective layer is used compared to no protective layer, but there is a capacity degradation due to the impedance evolution after 10th charge/discharge due to the poor intrinsic oxidation resistance of 400‐0.6B_12_‐0.4I SE (Figure S15, Supporting Information). A more suitable protective layer or other modifications need to be selected to improve the capacity degradation in future studies.

## 3 Conclusion

In summary, we successfully developed an innovative hydroborate‐based solid electrolyte (400‐0.6B_12_‐0.4I) through a synergistic dual‐modification strategy integrating high‐temperature phase engineering (400 °C, 2 h, in Ar) with LiI doping. Systematic characterization reveals that the thermal treatment induces a disordered high‐temperature phase structure in Li_2_B_12_H_12_, while LiI incorporation simultaneously optimizes ionic transport pathways and improves interfacial compatibility with lithium metal anodes. The resultant 400‐0.6B_12_‐0.4I SE demonstrates remarkable ionic conductivity of 0.75 mS cm^−1^ (the highest level among the reported inorganic Li_2_B_12_H_12_‐based solid electrolytes) at 25 °C, a threefold enhancement over pristine Li_2_B_12_H_12_, along with exceptional electrochemical stability evidenced by a high critical current density of 5.0 mA cm^−2^ that effectively suppresses lithium dendrite formation. These synergistic improvements enable unprecedented cycling stability in Li symmetric cells, maintaining ultralow polarization voltage (10 mV) over 1000 h of stable operation under practical current densities. Furthermore, 400‐0.6B_12_‐0.4I SE was coupled with various high‐voltage cathodes (LiCoO_2_, NCM811, and LiMn_2_O_4_) and lithium‐metal anodes. This combination exhibited favorable electrochemical performance, marking a significant advancement by demonstrating the feasibility of utilizing Li_2_B_12_H_12_‐based inorganic SEs in high‐voltage all‐solid‐state batteries. The solid electrolyte 400‐0.6B_12_‐0.4I still faces challenges of moisture absorption and high raw material costs, limiting its potential for mass production issues that require resolution in future research. Nevertheless, this work establishes a new paradigm in hydroborate electrolyte design, with the rationally engineered 400‐0.6B_12_‐0.4I composite emerging as a highly promising candidate for next‐generation all‐solid‐state lithium metal batteries.

## Conflict of Interest

The authors declare no conflict of interest.

## Supporting information



Supporting Information

## Data Availability

The data that support the findings of this study are available in the supplementary material of this article.
