# Supplementary material for: Facile Synthesis of Inorganic Li2B12H12/LiI Solid Electrolytes for High‐Voltage All‐Solid‐State Lithium Batteries
Source: Adv Sci (Weinh). 2025 Aug 29;12(43):e10193. doi: 10.1002/advs.202510193 (PMC12631882; doi:10.1002/advs.202510193)
Supplement: Supplementary file 1 — Supporting Information [file ADVS-12-e10193-s001.docx]

Supporting Information

**Facile Synthesis of Inorganic Li_2_B_12_H_12_/LiI Solid Electrolytes for High-Voltage All-Solid-State Lithium Batteries**

*Deliang Xu, Mengyuan Jin, Zilong Su, Ran Liu, Mingchuan Xiang,* *Wanggang Fang, Liqing He,* Renbing Wu, and Yanhui Guo**

**Supporting Text for Methods**

**Preparation of materials**

To synthesize Li_2_B_12_H_12_, the (Et_3_NH)_2_B_12_H_12_ was firstly synthesized according to previously published procedures^[1]^. Then, we added (Et_3_NH)_2_B_12_H_12_ in deionised water and stirred at 60 °C. LiOH solution was subsequently added dropwise until the pH of solution reached 7. The solvent was then removed by spin distillation and Li_2_B_12_H_12_ containing crystal water was obtained (Li_2_B_12_H_12_·xH_2_O). We further removed the water of crystallisation by vacuum heating to obtain anhydrous Li_2_B_12_H_12_. In addition, we have used different temperatures (300, 350, 400, 450 ℃) for the high temperature sintering of Li_2_B_12_H_12_ (named 300-Li_2_B_12_H_12_, 350-Li_2_B_12_H_12_, 400-Li_2_B_12_H_12_, 450-Li_2_B_12_H_12_). The compound of 400- Li_2_B_12_H_12_ and LiX (X=F, Cl, Br, I) at a molar ratio of 0.6:0.4 was ball-milled mechanically at 400 rpm for 5 h using a planetary mill to prepare 400-0.6Li_2_B_12_H_12_-0.4LiX (X=F, Cl, Br, I). Similarly, the compound of 400-Li_2_B_12_H_12_ and LiI in different molar ratio was ball-milled mechanically at 400 rpm for 5 h to prepare 400-xLi_2_B_12_H_12_-yLiI.

All the obtained samples were stored in the glove box (MIKROUNA, Universal, O_2_ and H_2_O content < 1 ppm) for further use.

**Materials characterizations.**

Due to the Li_2_B_12_H_12_-based SEs synthesized in this work are prone to absorb water, it's important to keep samples dry during all stages of characterization. The ^11^B and ^1^H solution-state NMR in CD_3_CN were conducted on a 500 MHZ NMR spectrometer (Bruker AVANCE III HD). The solid-state ^11^B and ^7^Li NMR experiments were recorded on a 400 MHz NMR spectrometer (Bruker AVANCE III 400WB). The SEs were put into special 3.2 mm zirconia rotors in the glove box that was filled with argon.

XRD patterns were recorded on Bruker D8 ADVANCE (Cu K*α*-radiation *λ* = 0.15405 nm, 40 kV, 40 mA). DSC measurements were carried out on HITACHI DSC200. Raman measurements were carried out on Horiba LabRAM HR Evolution. TG-MS measurements were carried out on TA SDT Q600. SE powders were loaded into a XRD holder and sealed with amorphous tapes to prevent the sample from being exposed to air and absorbing moisture during the test. FT-IR measurements were carried out on Nicolet Nexus 470 spectrometer in the wavelength range of 3000-400 cm^-1^. The samples were grounded into powders with KBr and pressed into thin pellets for measurement. The true density of Li_2_B_12_H_12_-based solid electrolyte was measured on JW-M100A Automatic true density analyzer under Helium atmosphere. Morphologies of the SE powder were observed using Phenom Prox scanning electron microscope (SEM). Transmission electron microscopy (TEM, Talos F200X) coupled with energy dispersive X-ray spectroscopy (EDX) were used for surface characterization and microstructural analysis.

**Electrochemical characterization.**

The electrochemical characterizations were performed on a CHI 760E electrochemical workstation. The Li-ion conductivities of SEs were calculated from electrochemical impedance spectroscopy (EIS) in the frequency of 10^6^~10^-1^ Hz with voltage amplitude of 5 mV. The SEs powders were pressed into thin sheets (thickness 0.4~0.6 mm, diameter 10 mm) under cold pressure of 480 Mpa for 0.5 h in a glove box and assembled into a blocking SS/SEs/SS cell using two stainless-steel bars as blocking electrodes and current collectors for measurement. The ionic conductivities (*σ*) of the SEs were calculated according to NernstEinstein equation (1) where *d* (cm) is the thickness of the SEs and *S* (cm^2^) is the area of the SEs. *R* (Ω) is the total resistance value of SEs:

𝜎 = 𝑑/(𝑅 × 𝑆) (1)

To gain the Arrhenius plots, EIS of SEs with different temperature from 30 to 100 ℃, the activation energies (*E_a_*) of SEs are then calculated base on the Nernst–Einstein equation and Arrhenius equation, where A_0_ is the pre-exponential factor and k is the Boltzmann constant:

𝜎𝑇 = A_0_ 𝑒𝑥𝑝( -E_a_/kT) (2)

The electronic conductivity was calculated using equation (3),which was obtained from the DC polarization curve of the blocking SS/SEs/SS cell ,V (v) is the applied DC voltage of 1 V, i_ss_ (A) is the steady-state current after 10000 s, *d* (cm) is the thickness of the SEs and *S* (cm^2^) is the area of the SEs :

𝜎_i_ = 𝑑/S(V × i_ss_) (3)

The Li-ion transference number (*t*_Li+_) of the SEs was determined by the Bruce–Vincent method. The symmetric Li/SEs/Li cell was assembled and a 10 mV polarization potential was applied to the cell until current kept steady. The corresponding EIS before and after the DC polarization were recorded. *t*_Li+_ was measured according to the following equation, where *I*_0_ and *I*_ss_ (A) are the initial and steady current, respectively. *ΔV* is the applied voltage (V), *R_0_* and *R_ss_* (Ω) are the resistance recorded from Nyquist plots before and after DC polarization, respectively:

t_Li+_ = 𝐼_ss_(∆𝑉 ― 𝐼_0_𝑅_0_)/𝐼_0_(∆𝑉 ― 𝐼_𝑠𝑠_𝑅_𝑠𝑠_) (4)

**Cell assembly and electrochemical measurement.**

All cells were assembled in an Ar-filled glove box with O_2_ and H_2_O below 0.01 ppm. The symmetric cell Li/SE/Li was assembled to study the Li plating/striping performance, which was reassembled by attaching Li plate on each side of the pressed SE sheet. The galvanostatic cycles were recorded to evaluate the compatibility of the SEs towards Li metal using constant-current mode. The critical current density (CCD) was determined from the current of the last cycle before the potential drop by gradually increasing the current from 0.05 mA cm^-2^ per cycle.

The LiCoO_2_/Li_3_InCl_6_/400-0.6B_12_-0.4I/Li, LiMn_2_O_4_/Li_3_InCl_6_/400-0.6B_12_-0.4I/Li and NCM811/Li_3_InCl_6_/400-0.6B_12_-0.4I/Li all-solid-state battery were assembled according to the following process: Firstly, 80 mg 400-0.6B_12_-0.4I powder was pressed into thin sheet (diameter 10 mm) under 420 MPa the composite cathodes were prepared by manually grinding the cathode material. Secondly, 40 mg Li_3_InCl_6_ was spread all over the thin sheet and pressed under the same pressure again. Thirdly, the composite cathode were prepared by mixing cathode materials (LiCoO_2_, LiMn_2_O_4_ and NCM811), Li_3_InCl_6_ and carbon black in the weight ratio of 70:20:10 using a mortar and pestle. Next, 5 mg of the composite cathode was evenly distributed onto Li_3_InCl_6_ side of the densified electrolyte sheet and pressed at 50 MPa for 2 min. Finally, a piece of Li foil was attached on the 400-0.6B_12_-0.4I side of the electrolyte layer as the anode. All the measurements mentioned above were performed using a LAND CT2001A battery testing system.

**Computational method:**

AIMD calculations were carried out by using spin-polarized DFT with the generalized gradient approximation (GGA) and Perdew-Burke-Ernzerhof (PBE) as implemented in the Vienna ab initio simulation package (VASP). A 2×2×1 supercell of Li_2_B_12_H_12_ were generated from the Material Project database that contained 432 atoms. The plane-wave energy cutoff was set to 500 eV for all calculations. The convergence threshold was set at 10^-5^ eV for the iteration in self-consistent field (SCF). The van der Waals interactions were described using the empirical correction in DFT-D2. For all AIMD simulations, a time step of 1 fs was used to integrate the equations of motion. The modified structural model was generated using Parrinello-Rahman dynamics with variable cell shape and volume, where the temperature was controlled using a Langevin thermostat. First, the structure was heated to 400 °C in 5 ps, and then doped with LiI to continuously melt 5ps. The structure was then cooled to 25 °C in 5 ps, followed by equilibration for 2 ps at 25 °C. DFT calculations were used to find the minimum energy structure. The canonical (NVT) ensemble was used to simulate the Li diffusion at 327 °C. The structure was first heated to 327 °C in 5 ps. Finally, the simulation was continued for 20 ps with NVT ensemble to collect data.


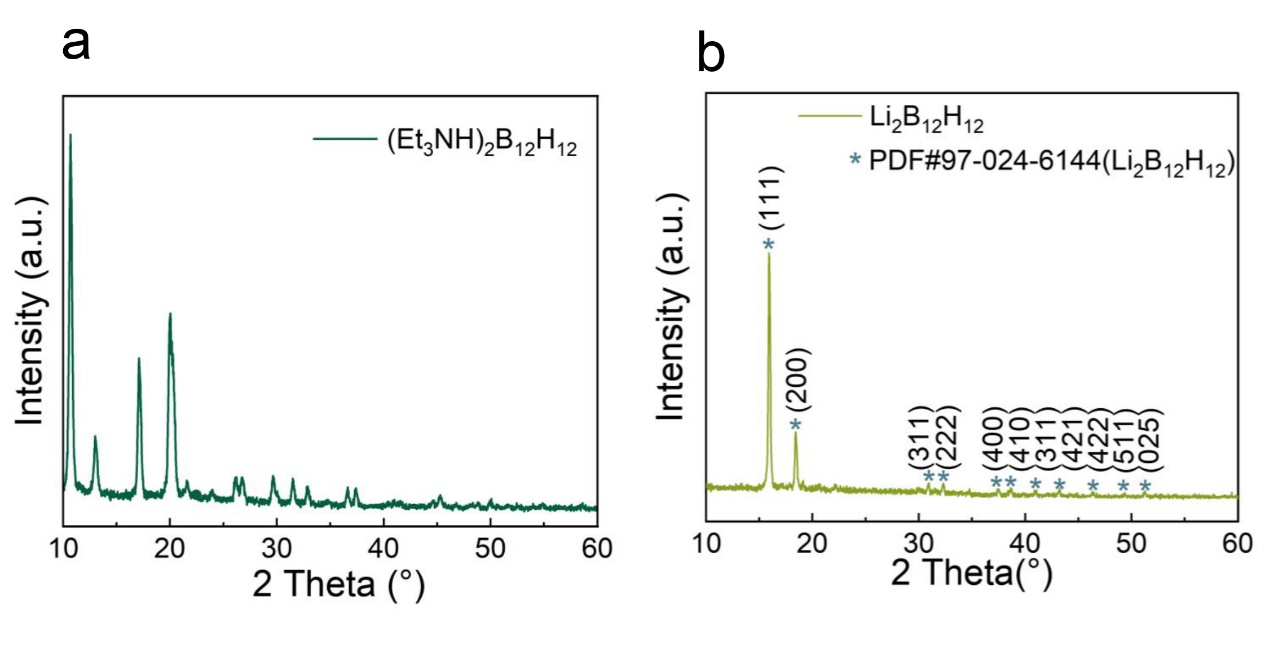


**Figure S1.** XRD pattern of the (Et_3_NH)_2_B_12_H_12_ (a) and Li_2_B_12_H_12_ (b) powder.


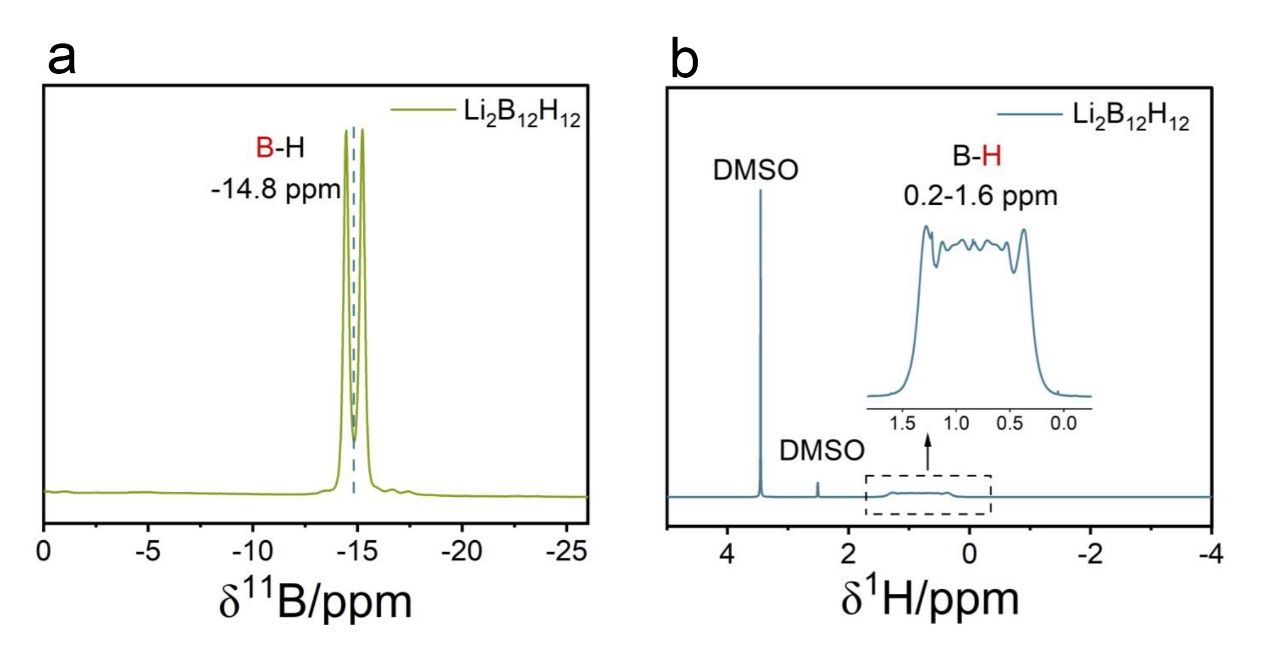


Fi**gure S2**. Liquid-state ^11^B NMR spectra (a) and ^1^H NMR spectra (b) of Li_2_B_12_H_12_.


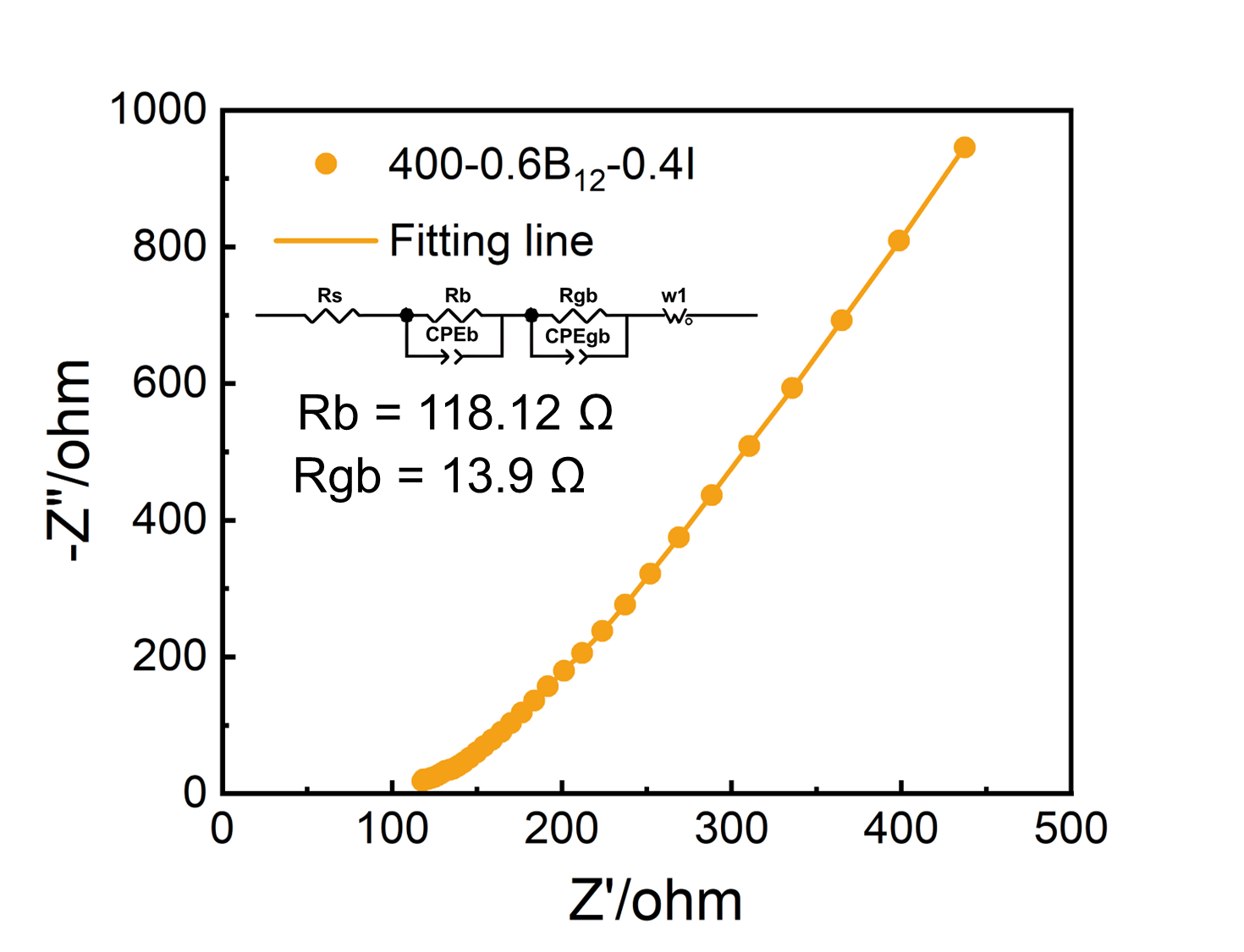


**Figure S3.** Nyquist plots of the 400-0.6B_12_-0.4I at 25 °C fitted with an equivalent circuit shown in the inset.


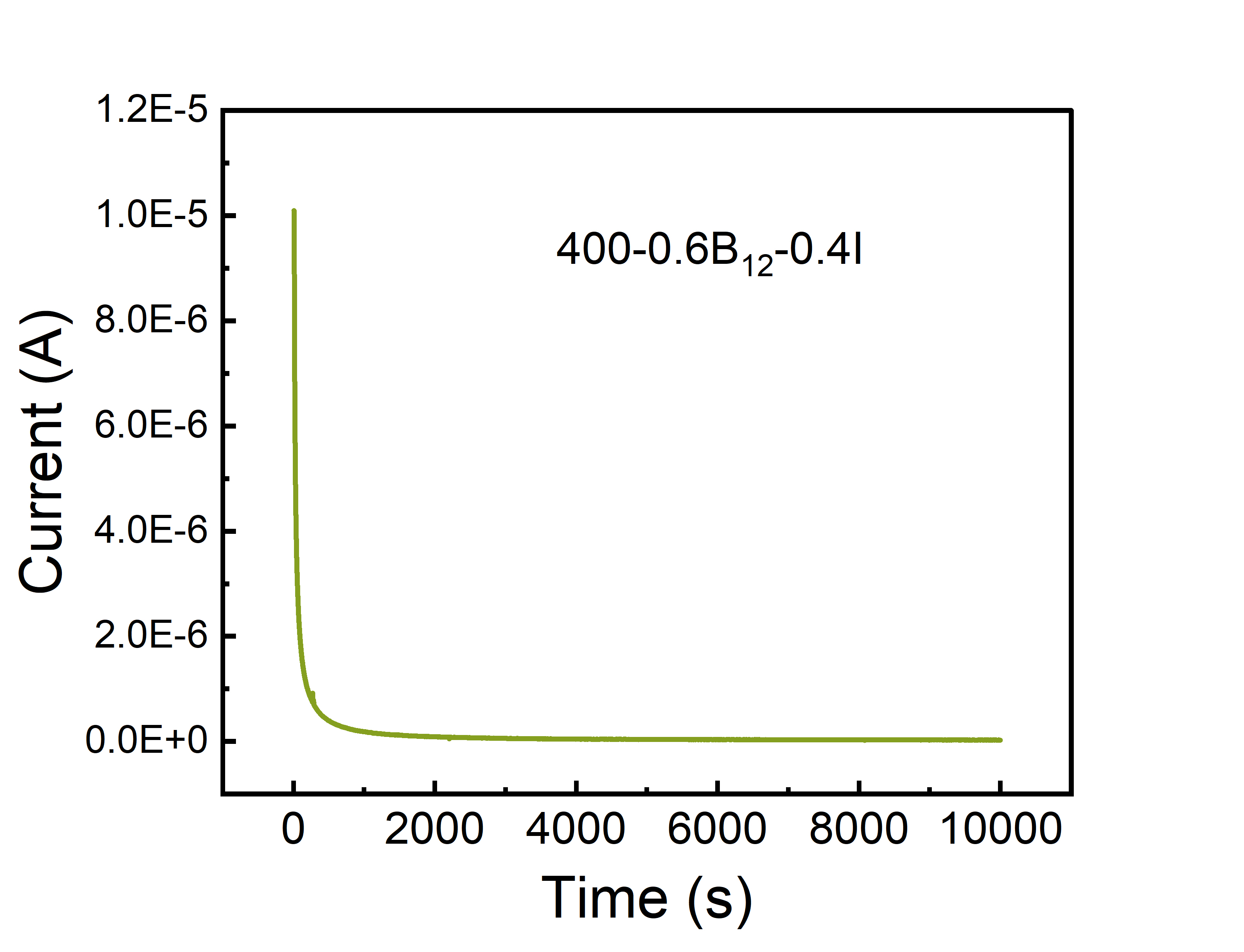


**Figure S4.** Electronic conductivity of 400-0.6B_12_-0.4I SEs.


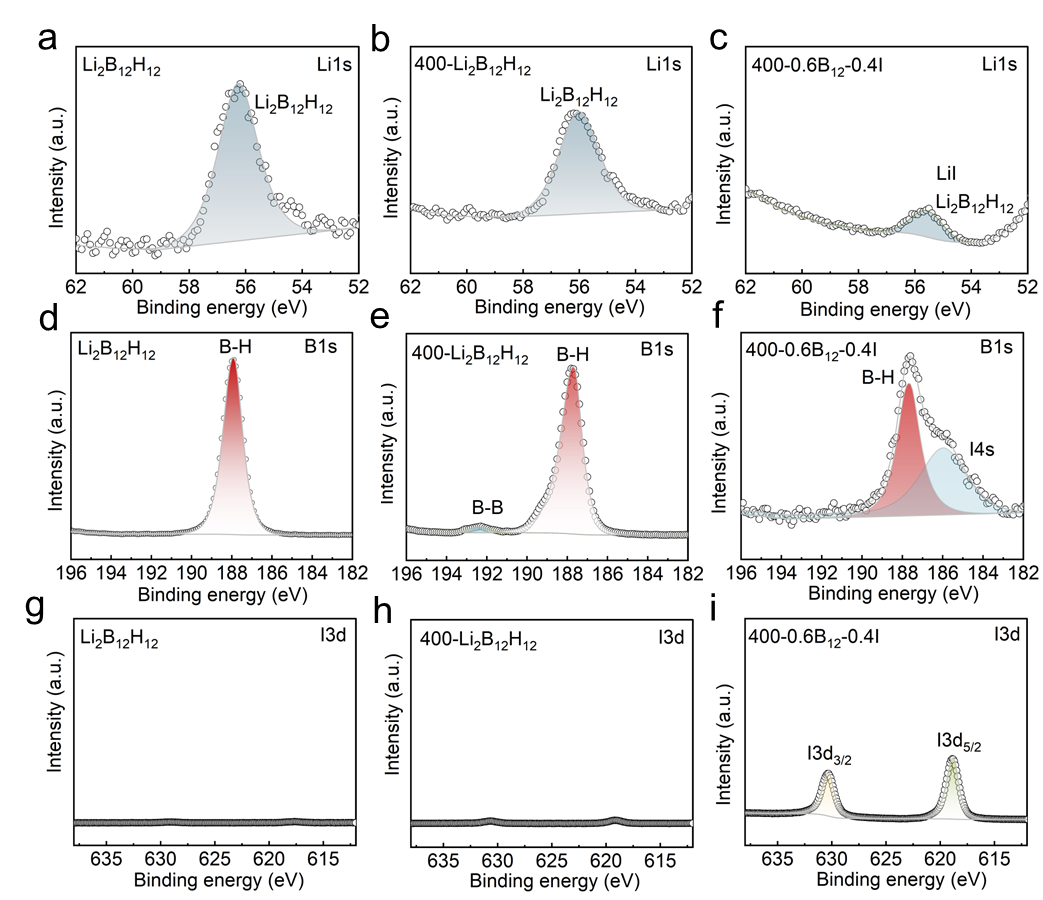


**Figure S5.** XPS analysis of Li_2_B_12_H_12_, 400-Li_2_B_12_H_12_ and 400-0.6B_12_-0.4I SEs.


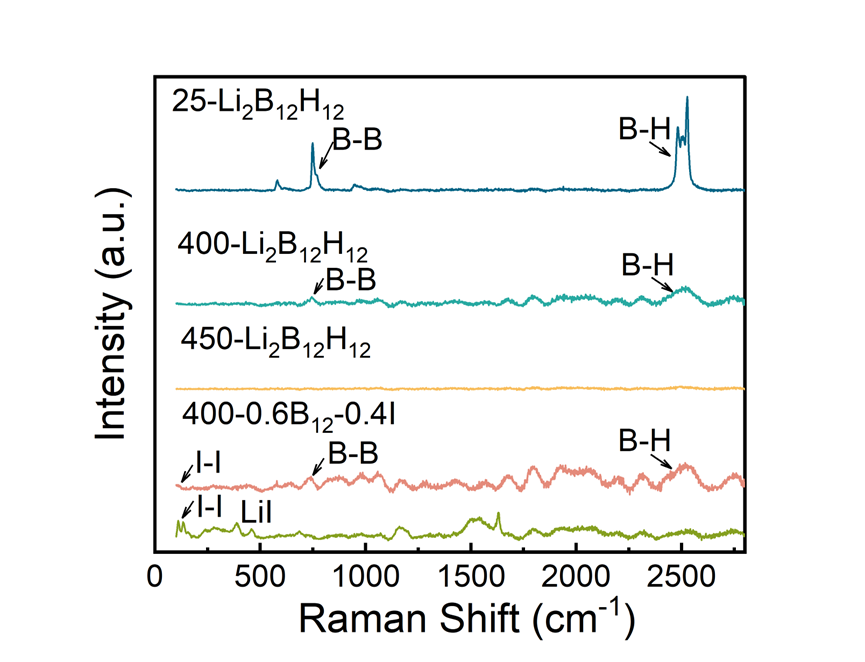


**Figure S6.** Raman spectra of 25-Li_2_B_12_H_12_, 400-Li_2_B_12_H_12_, 450-Li_2_B_12_H_12_, 400-0.6B_12_-0.4I and LiI.


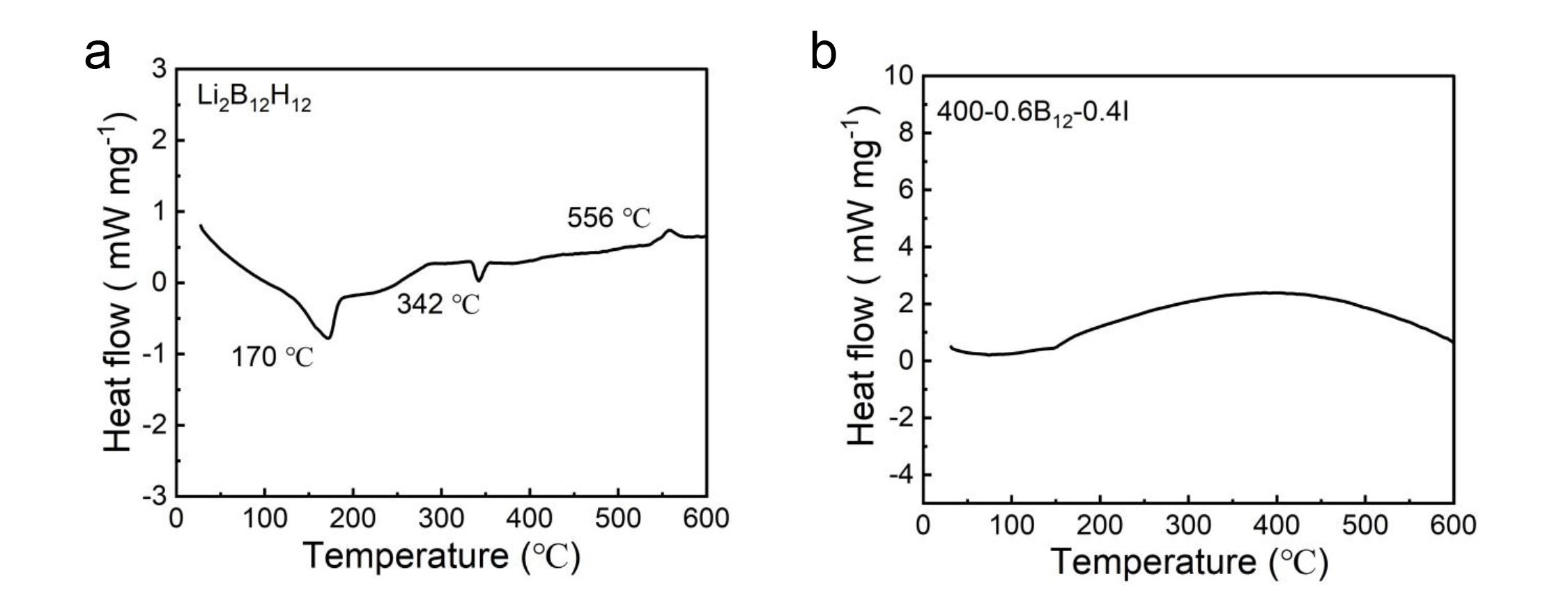


**Figure S7.** DSC spectra of Li_2_B_12_H_12_ and 400-0.6B_12_-0.4I.


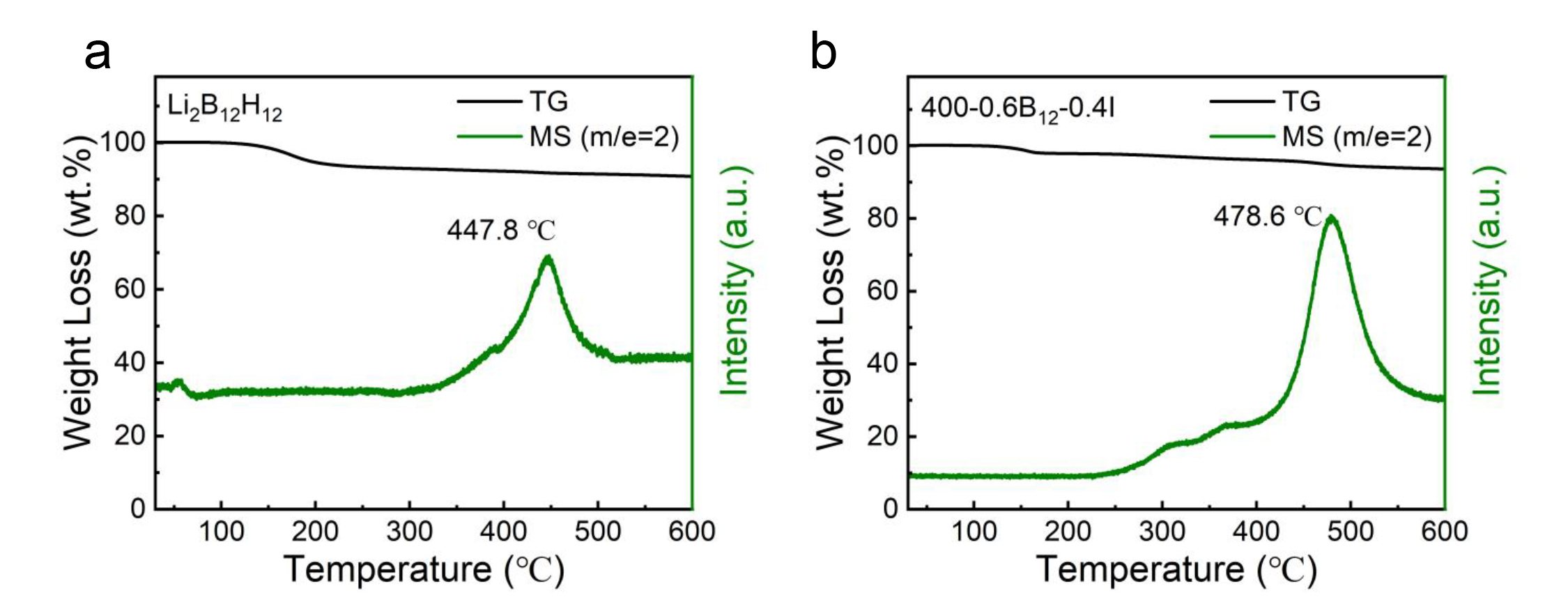


**Figure S8.** TG/MS spectra of Li_2_B_12_H_12_ and 400-0.6B_12_-0.4I.


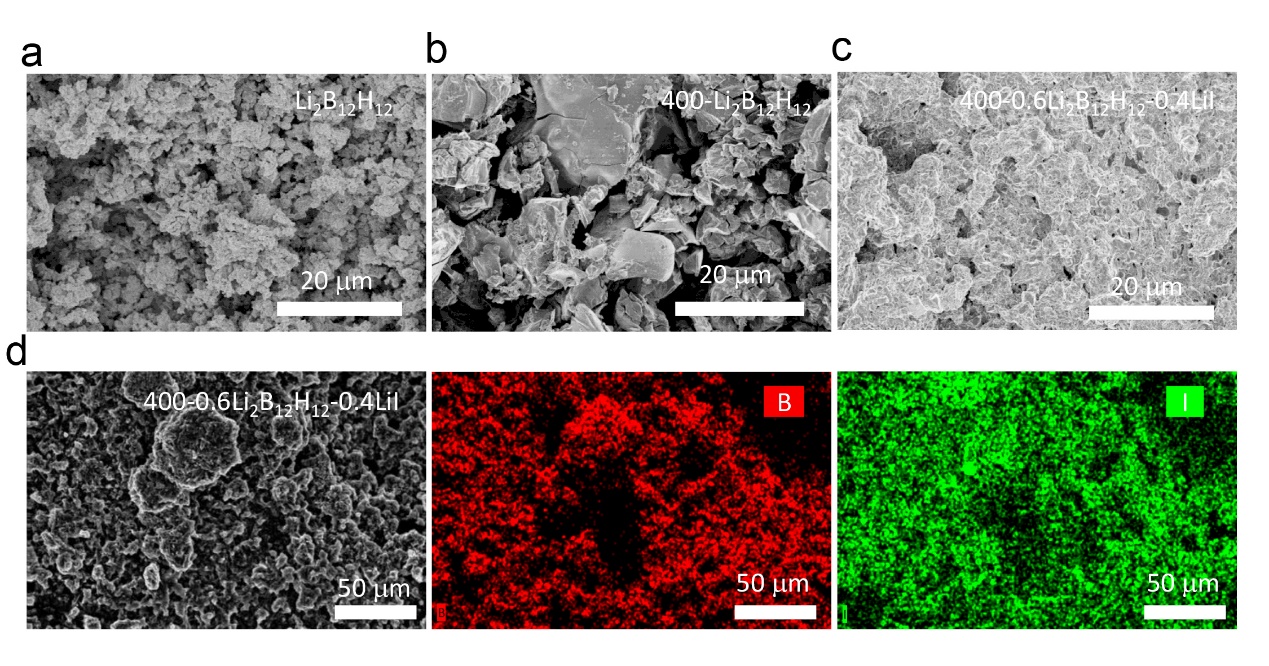


**Figure S9.** SEM image of (a) Li_2_B_12_H_12_, (b) 400-Li_2_B_12_H_12_, (c) 400-0.6B_12_-0.4I.


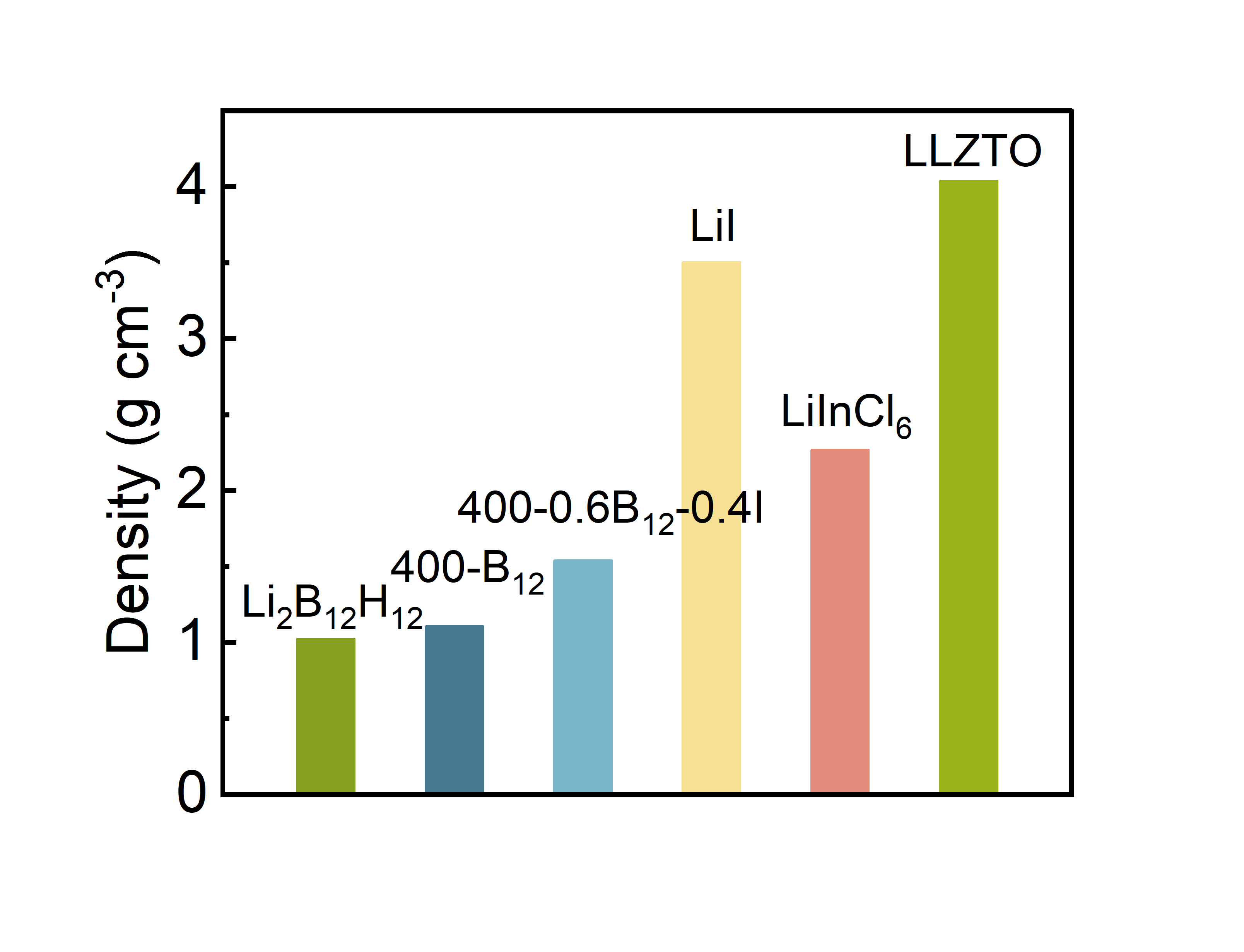


**Figure S10.** Comparison of 400-0.6B_12_-0.4I with various solid-state electrolytes and lithium salt densities.


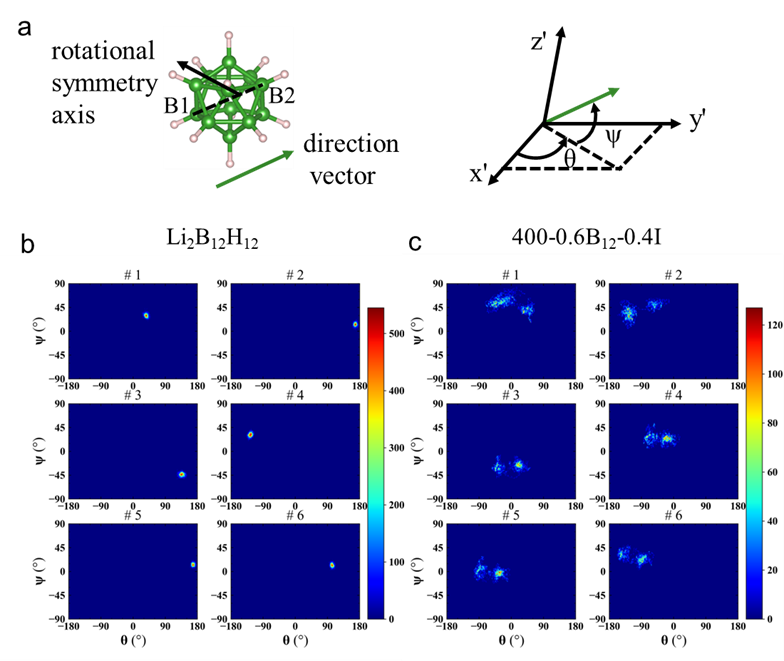


**Figure S11.** (a) The direction vector of a B_12_H_12_ group and the azimuth and elevation angles (*θ, ψ*) used in the calculation. The angles are defined in the local coordinate frame (x′, y′, z′) while the simulation box is set in (x, y, z). Angular pseudo-density map of multiple representative B12H12 groups of b) Li_2_B_12_H_12_ and c) 400-0.6B_12_-0.4I.


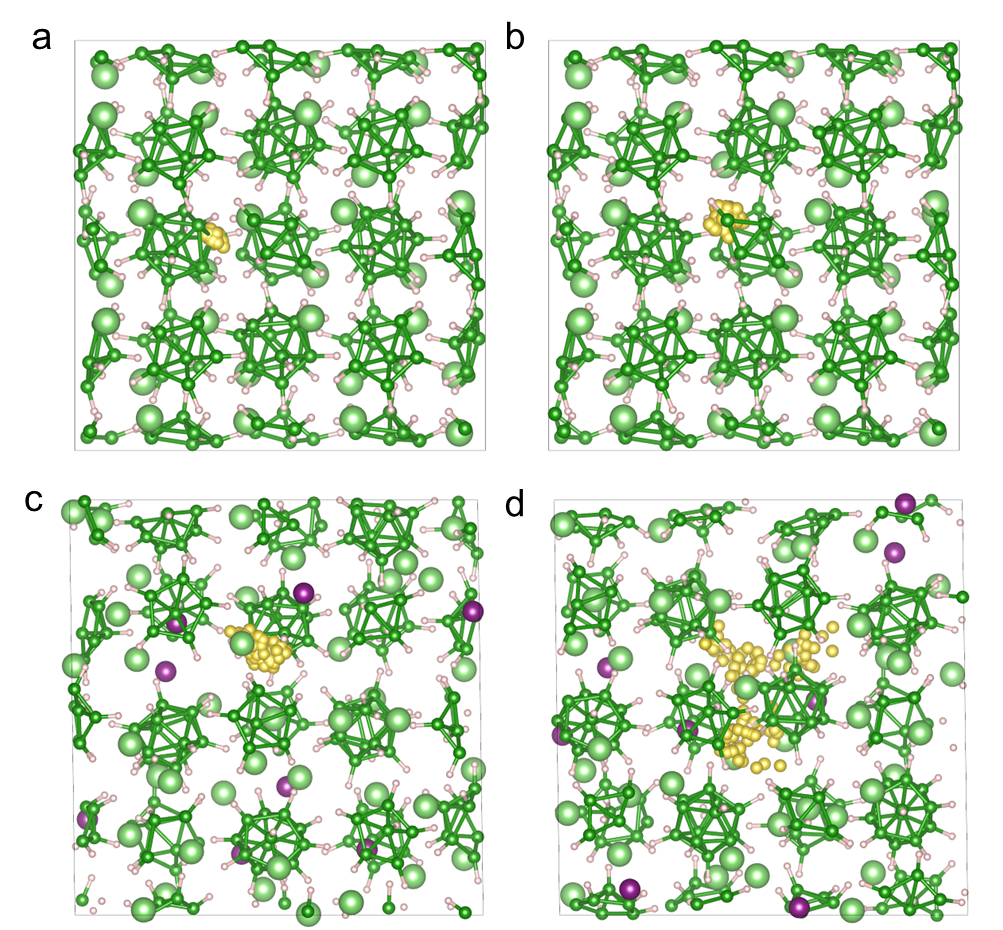


**Figure S12**. AIMD calculation for tracking the trajectory of individual B and Li atoms. Real-time trajectory for selected B atoms (a) and Li atoms (b) of Li_2_B_12_H_12_, and B atoms (c) and Li atoms (d) of 400-0.6B_12_-0.4I over a 20 ps time interval.


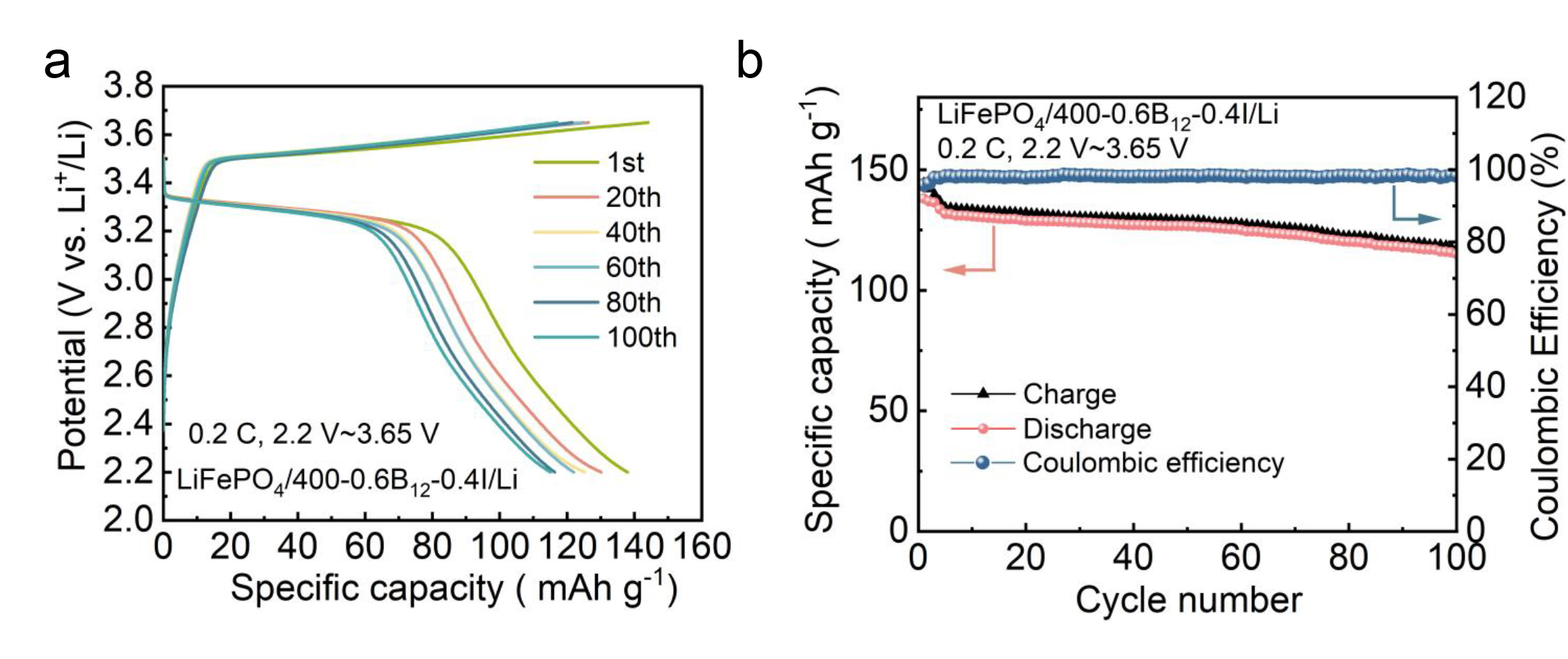


**Figure S13.** All-solid-state battery performance with LiFePO_4_ cathode of the 400-0.6B_12_-0.4I SE. (a) Corresponding GDC curves, (b) cycle performance of Li/400-0.6B_12_-0.4I/ LiFePO_4_.


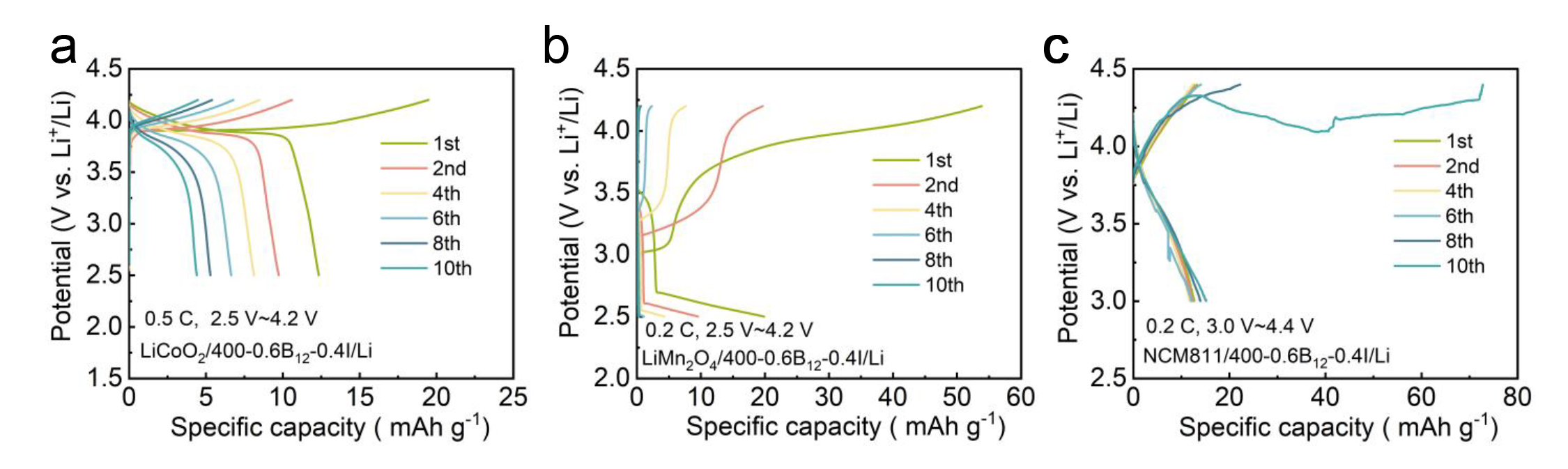


**Figure S14.** All-solid-state battery performance with high voltage cathodes of the 400-0.6B_12_-0.4I SSE without Li_3_InCl_6_ layer. GDC curves (a) of Li/400-0.6B_12_-0.4I/LiCoO_2_, Li/400-0.6B_12_-0.4I/LiMn_2_O_4_ and Li/400-0.6B_12_-0.4I/NCM811.


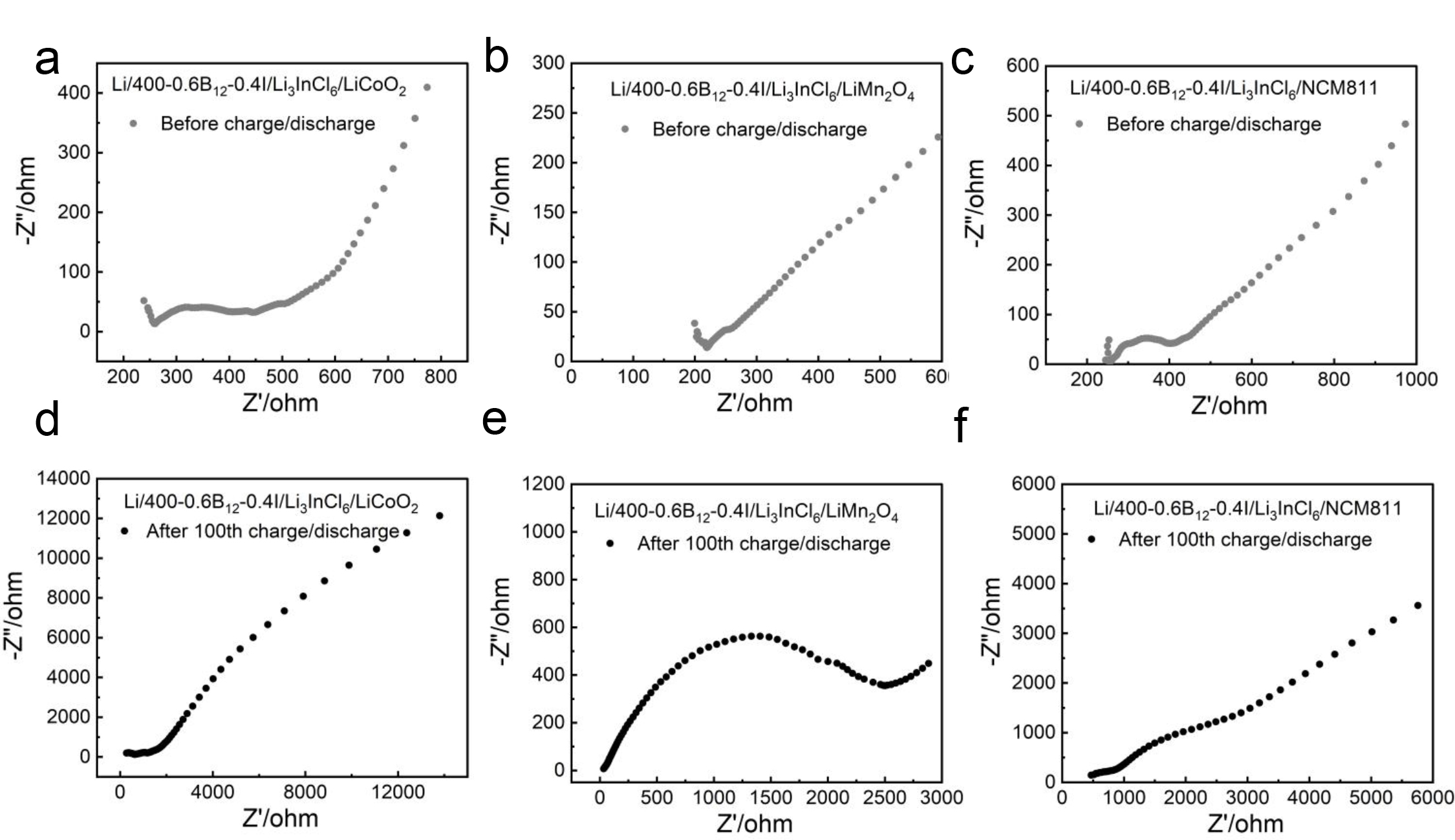


**Figure S15.** The impedance evolution before and after charge/discharge of Li/400-0.6B_12_-0.4I/Li_3_InCl_6_/LiCoO_2_, Li/400-0.6B_12_-0.4I/Li_3_InCl_6_/LiMn_2_O_4_ and Li/400-0.6B_12_-0.4I/Li_3_InCl_6_/NCM811.

**Table S1.** Comparison of the ionic conductivity and symmetric cells performance of 400-0.6B_12_-0.4I with different borohydride solid electrolytes.

| Solid electrolytes | Ionic conductivity | CCD | Li/Li | Ref |
| --- | --- | --- | --- | --- |
| 400-0.6B_12_-0.4I | 0.75 mS cm^-1^ at 25 °C | 5.0 mA cm^-2^ at 25 °C;  7.5 mA cm^-2^ at 60 °C. | 1000 h at  0.1 mA cm^-2^ | This work |
|  |  |  | 2000 h at  0.4 mA cm^-2^ |  |
| Li_2_B_12_H_12_ | 0.31 mS cm^-1^ at 60 °C | \ | \ | [2] |
| atom-deficient Li_2_B_12_H_12_ | 0.02 mS cm^-1^ at 30 °C | \ | \ | [3] |
| Li-B-H Complex | 0.27 mS cm^-1^ at 35 °C | \ | 1000 h at  1 mA cm^-2^, 30 °C | [4] |
| Li_4_B_10_H_10_B_12_H_12_ | 0.4 mS cm^-1^ at 25 °C | \ | \ | [5] |
| LiF decorated Li_2_B_12_H_12_ | 0.5 mS cm^-1^ at 75 ℃ | 3.6 mA cm^-2^ at 75℃ | \ | [6] |
| Li_2_B_12_H_12_-5Li_2_B_10_H_10_-6LiBH_4_ | 0.1 mS cm^-1^at room temperature | 1.0 mA cm^-2^ at room temperature | 120 h at  0.2 mA cm^-2^ | [7] |
| PEO-LiB_12_H_12_-PVDF-DMF | 0.465 mS cm^-1^ at 25 °C | \ | \ | [8] |
| PEO-Li_2_B_12_H_12_-LiTFSI-NMP | 0.143 S cm^-1^ | 0.2 mA cm^-1^ | 1200 h at  0.2 mA cm^-2^ | [9] |
| Li-B-H-Se-0.14 | 0.16 mS cm^-1^ at 60 °C | \ | \ | [10] |
| LLZTO-*x*LiBH_4_ | 0.0802 S cm^-1^ at 30 °C | 0.15 mA cm^-2^ at 30 °C | 300 h at  0.15 mA cm^-2^ | [11] |
| LiBH_4_-Al_2_O_3_ | 0.32 mS cm^-1^at 75 ℃ | \ | \ | [12] |
| LiBH_4_-MgO | 0.286 mS cm^-1^at 20 ℃ | \ | 90 h at  0.025 mA cm^-2^ | [13] |

**Table S2.** Comparison of the ASSBs performance of 400-0.6B_12_-0.4I with different inorganic Li_2_B_12_H_12_-based solid electrolytes.

| Solid electrolytes | Cathode/anode | Voltage/V | Cycle number | Rate performance | Ref |
| --- | --- | --- | --- | --- | --- |
| 400-0.6B_12_-0.4I | LiCoO_2_/Li | 2.5-4.2 | 0.5C, 100 | 2C | This work |
|  | LiMn_2_O_4_/Li | 2.5-4.2 | 0.2C, 100 | 1C |  |
|  | NCM811/Li | 3.0-4.4 | 0.2C, 100 | 1C |  |
| Li_2_B_12_H_12_ | TiS_2_/Li | 1.6-2.7 | 0.2C, 10 | 0.2C | [2] |
| Li_4_B_10_H_10_B_12_H_12_ | TiS_2_/Li | 1.6-2.5 | \ | 1C | [5] |
|  | LiFePO_4_/Li | 2.0-3.6 | \ | 1C |  |
| LiF decorated LiB_12_H_12_ | LiFePO_4_/Li | 2.1-4.0 | 30 | \ | [6] |
| Li_2_B_12_H_12_-5Li_2_B_10_H_10_-6LiBH_4_ | TiS_2_/In_1.3_Li_0.3_ | 1.8-2.7 | 0.1C, 120 | \ | [7] |

**Table S3.** The area capacities and N/P values of full-cell.

| Full-cell type | Areal capacity | N/P ratio |
| --- | --- | --- |
| Li/400-0.6B_12_-0.4I/Li_3_InCl_6_/LiCoO_2_ | 1.73 mAh cm^-2^ | 5.95 |
| Li/400-0.6B_12_-0.4I/Li_3_InCl_6_/LiMn_2_O_4_ | 1.67 mAh cm^-2^ | 6.17 |
| Li/400-0.6B_12_-0.4I/Li_3_InCl_6_/NCM811 | 2.02 mAh cm^-2^ | 5.10 |

**Reference**

[1] J. M. Makhlouf, W. V. Hough, G. T. Hefferan, Practical synthesis for decahydrodecaborates, *Inorg. Chem.* **1967**, *6*, 1196-1198.

[2] A. Unemoto, K. Yoshida, T. Ikeshoji, S.-i. Orimo, Bulk-Type All-Solid-State Lithium Batteries Using Complex Hydrides Containing Cluster-Anions, *Mater. Trans.* **2016**, *57*, 1639-1644.

[3] S. Kim, N. Toyama, H. Oguchi, T. Sato, S. Takagi, T. Ikeshoji, S.-i. Orimo, Fast Lithium-Ion Conduction in Atom-Deficient closo-Type Complex Hydride Solid Electrolytes, *Chem. Mater.* **2018**, *30*, 386-391.

[4] M. Zhu, Y. Pang, F. Lu, X. Shi, J. Yang, S. Zheng, In Situ Formed Li-B-H Complex with High Li-Ion Conductivity as a Potential Solid Electrolyte for Li Batteries, *ACS Appl. Mater. Interf.* **2019**, *11*, 14136-14141.

[5] A. Garcia, G. Müller, R. Černý, D. Rentsch, R. Asakura, C. Battaglia, A. Remhof, Li_4_B_10_H_10_B_12_H_12_ as solid electrolyte for solid-state lithium batteries, *J. Mater. Chem. A* **2023**, *11*, 18996-19003.

[6] X. Shi, Y. Pang, B. Wang, H. Sun, X. Wang, Y. Li, J. Yang, H. W. Li, S. Zheng, In situ forming LiF nanodecorated electrolyte/electrode interfaces for stable all-solid-state batteries, *Mater. Today Nano* **2020**, *10*, 100079.

[7] C. Zhou, Y. Yan, T. R. Jensen, Enhanced Electrochemical Performance of the Li_2_B_12_H_12_-Li_2_B_10_H_10_-LiBH_4_ Electrolyte, *ACS Appl. Energy Mater.* **2023**, *6*, 7346-7352.

[8] X.-Y. Ye, K.-P. Bao, S.-N. Luo, X. Li, T.-Q. Chen, S.-X. Xia, T. Yuan, Y.-P. Pang, S.-Y. Zheng, Evolution and function of residual solvent in polymer-Li_2_B_12_H_12_ composite solid electrolyte, *Rare Metals* **2024**, 3748–3757

[9] K. Bao, Y. Pang, J. Yang, D. Sun, F. Fang, S. Zheng, Modulating composite polymer electrolyte by lithium closo-borohydride achieves highly stable solid-state battery at 25°C, *Sci. China Mater.* **2021**, *65*, 95-104.

[10] Y. Yin, F. Yan, S. Li, Y. Chen, D. Guo, J. Zhao, D. Sun, F. Fang, Y. Song, Nature‐Inspired Strategy: Novel Borohydride‐Based Solid Electrolytes Extracted from Cathode‐Electrolyte Interphase, *Adv. Mater.* **2024**, *36,* 2406632

[11] Y. Gao, S. Sun, X. Zhang, Y. Liu, J. Hu, Z. Huang, M. Gao, H. Pan, Amorphous Dual‐Layer Coating: Enabling High Li‐Ion Conductivity of Non‐Sintered Garnet‐Type Solid Electrolyte, *Adv. Funct. Mater.* **2021**, *31*, 2009692

[12] S. Zeng, K. Ren, H. Ding, S. Xu, H.-W. Li, Y. Li, Stabilization of fast lithium-ionic conduction phase of nanaoconfined LiBH4 for lithium metal solid-state batteries, *Chem. Commun.* **2025**, *61*, 929-932.

[13] J. B. Grinderslev, L. N. Skov, J. G. Andreasen, S. Ghorwal, J. Skibsted, T. R. Jensen, Methylamine Lithium Borohydride as Electrolyte for All‐Solid‐State Batteries, *Angew. Chem. Int. Ed.* **2022**, *61*, e202203484.
